# Environmental Noise, Genetic Diversity and the Evolution of Evolvability and Robustness in Model Gene Networks

**DOI:** 10.1371/journal.pone.0052204

**Published:** 2012-12-20

**Authors:** Christopher F. Steiner

**Affiliations:** Department of Biological Sciences, Wayne State University, Detroit, Michigan, United States of America; University of California Santa Barbara, United States of America

## Abstract

The ability of organisms to adapt and persist in the face of environmental change is accepted as a fundamental feature of natural systems. More contentious is whether the capacity of organisms to adapt (or “evolvability”) can itself evolve and the mechanisms underlying such responses. Using model gene networks, I provide evidence that evolvability emerges more readily when populations experience positively autocorrelated environmental noise (red noise) compared to populations in stable or randomly varying (white noise) environments. Evolvability was correlated with increasing genetic robustness to effects on network viability and decreasing robustness to effects on phenotypic expression; populations whose networks displayed greater viability robustness and lower phenotypic robustness produced more additive genetic variation and adapted more rapidly in novel environments. Patterns of selection for robustness varied antagonistically with epistatic effects of mutations on viability and phenotypic expression, suggesting that trade-offs between these properties may constrain their evolutionary responses. Evolution of evolvability and robustness was stronger in sexual populations compared to asexual populations indicating that enhanced genetic variation under fluctuating selection combined with recombination load is a primary driver of the emergence of evolvability. These results provide insight into the mechanisms potentially underlying rapid adaptation as well as the environmental conditions that drive the evolution of genetic interactions.

## Introduction

In his classic studies of evolution in heterogeneous environments, Levins proposed that a population’s reduction in mean fitness due to temporal environmental variation can be alleviated if the temporal covariance between its mean phenotype and the changing optimal phenotype is sufficiently large [1], [2]. The ability to track environmental change depends on the presence of additive genetic variance. However, genetic variance also reduces fitness by introducing non-optimal phenotypes to a population. Thus, a genetic response to a changing environment will only be favored if it is rapid and accurate enough such that covariances compensate for any fitness losses due to phenotypic variation. In weakly autocorrelated or randomly varying environments there is little opportunity for populations to accurately track environmental variation and selection for variant phenotypes is weak. Only when environmental fluctuations exhibit a sufficient degree of autocorrelation and predictability through time (i.e., when fluctuations are “reddened” or “red shifted”) will the presence of additive genetic variance and tracking of environmental change be at a selective advantage [1], [2].

**Figure 1:**
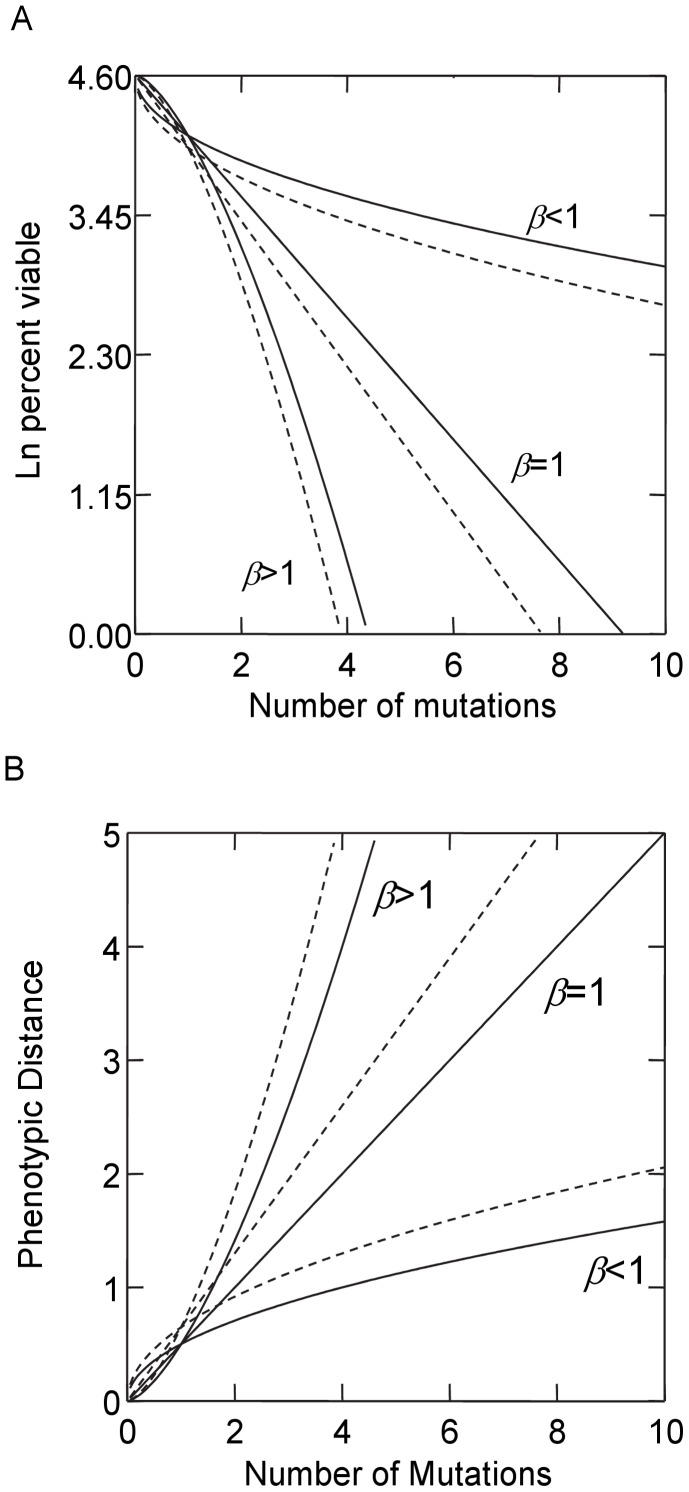
Hypothetical relationships between correlates of fitness (*W_m_*) and number of mutations (*m*). (**A**) Effects of lethal mutations measured as the natural log of the percent of mutants that are viable (*W_m_*); curves were generated using the relationship ln(*W_m_*) = −*αm^β^*+ ln(100). (**B**) Effects of mutations on the phenotypic distance between the mutant and wild type; curves were generated using *W_m_* = *αm^β^*. For both A and B, *β* measures the strength and direction of epistasis. *β*>1 indicates negative directional (or synergistic) epistasis in which each subsequent mutation has a greater effect than the last, *β*<1 indicates positive directional (or antagonistic) epistasis in which effects of mutations become weaker as they accumulate, and *β* = 1 indicates effects of mutations are the same as they accumulate. Each dashed lines corresponds to larger *α* and stronger effects of mutations relative to the paired solid line.

One potential facet of a genetic system’s ability to generate additive genetic variation is its capacity to buffer the deleterious effects of mutations (what is commonly termed genetic canalization); genomes that reduce the lethality of mutations may be better able to produce phenotypic variation from mutational events, effectively exploring a greater proportion of genotypic/phenotypic space without suffering lethal effects from mutations [3]–[7]. Hence, the evolution of evolvability may be coupled with the capacity of genetic buffering mechanisms to respond to selection. The ability to buffer phenotypic responses to genetic change (whether via mutation, recombination or gene flow) may include two interdependent properties: genetic robustness (the overall magnitude of the effect of mutations) and the strength and direction of epistatic interactions (the degree to which the effects of mutations are dependent on the presence of other genes). Figure 1A displays both properties as measured by the effects of mutation accumulation on the log percentage of viable phenotypes (*W_m_*) produced by a given genotype. The scaling of this relationship, determined by the parameter *β*, corresponds to different forms of epistasis (see Fig. 1 legend). For a given *β*, robustness is represented as differences in the overall (or average) effects of mutations on the percent viable and is inversely related to the normalization constant (*α*) [8].

While the capacity to maintain viability in the face of accumulating mutations is one important aspect of robustness, robustness can also be measured as the effect of mutations on phenotypic expression relative to the pre-mutation phenotype. This definition of robustness is similar to prior treatments of genetic canalization and the ability of genetic systems to suppress phenotypic variation caused by mutations [9]. Greater robustness in this sense may actually lead to a decrease in a genotype’s inherent evolvability by diminishing its capacity to produce variant offspring phenotypes [6]. Similarly, epistatic effects on phenotypic expression could also influence evolvability; genotypes that exhibit synergistic epistatic effects on phenotypic expression can produce phenotypes that differ much more from the wild type as mutations accumulate compared to those that exhibit antagonistic epistasis (Fig. 1B).

Prior theoretical investigations have shown that robustness and epistasis can readily evolve [4], [10]–[13]. Selection for robustness may be particularly strong when populations harbor a large amount of genetic variation - a byproduct of several potential factors including large population size, high mutation rates and recombination [9]. Sexual reproduction, when combined with genetic variation, can impose a recombination load on populations by disassembling favorable gene combinations and producing deleterious ones, thus selecting for enhanced robustness. While strong stabilizing selection can counter such effects by reducing genetic variation within populations [9], temporal variation may in theory strengthen selection for robustness by promoting diversifying selection and genetic variation [14]–[16]. Thus, autocorrelated temporal variation may select for evolvability via direct and indirect pathways that feedback on each other - directly by favoring genotypes that produce highly variant offspring and indirectly by promoting the maintenance of genetic/phenotypic variation which then selects for robustness.

The conditions that give rise to the evolution of robustness and directional epistasis are an area of active debate and research [5], [7], [10], [17]. Whereas prior studies have shown that mutations that affect fitness commonly interact with each other and their genetic backgrounds [18]–[20], an understanding of the form and prevalence of directional epistasis in nature remains a major challenge in evolutionary biology [18]. This is especially important as directional epistasis can, in theory, influence numerous evolutionary processes including reproductive isolation, the nature of the adaptive process (sensu Wright’s shifting balance theory) and the evolution of sexual reproduction [20]. One potential but little studied force of change that may shape epistatic interactions and robustness is temporally varying environmental conditions [21], [22]. Although prior studies have shown that changing evolutionary optima can influence the adaptive capacity of model genetic systems [4], [23], few have explicitly examined the role of colored environmental noise and the links between the maintenance of genetic variation, selection for robustness/epistasis, and the evolution of evolvability. Understanding the evolutionary consequences of autocorrelated environmental variation is especially important in light of the increasing recognition that environmental fluctuations in nature are frequently red-shifted (e.g., temperature and precipitation) and that natural population fluctuations also commonly exhibit positive autocorrelation [24]–[26]. Moreover, anthropogenic effects on climate change may include effects on the autocorrelational structure of environmental conditions such as temperature [27]. Thus, comprehending how the color spectra of environmental variation impact the adaptive capacity of genetic systems is an important component of predicting future impacts on natural systems. To address these questions I explored the effects of environmental fluctuations on the evolution of model gene networks, building off of a prior model framework [9], [10], [12], [23], [28]–[30]. In this individual-based model, each genotype in a population is represented by a matrix of interacting genes (or transcriptional regulators). Populations of gene networks were allowed to evolve for 36000 generations (with recombination and mutation) under four different selection regimes: stabilizing (no variation), directional, white noise (random temporal variation), or reddened noise (positively autocorrelated temporal variation). By combining explicit genetic interactions with population and evolutionary processes this model allowed exploration of environmental effects on the evolution of emergent genetic properties as well as the mechanisms underlying such responses.

## Methods

### General Model Framework

Each genotype in a population was represented by a *N* x *N* matrix (*A*) of *N* interacting genes (or transcriptional regulators) with *N* set at 10 for all simulations. The proportion of non-zero elements in *A* characterizes its connectance (*c*) which was set equal to 0.75 - chosen to facilitate comparisons with prior studies [10], [12], [28]. However, preliminary results using connectance levels of 0.20 and 0.85 produced patterns qualitatively similar to those presented here (Fig. S1). An individual’s gene expression levels during development are contained in the vector **S**(*t*) and is determined for each gene *i* at time t+1 by:
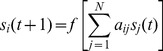
(1)where *a_ij_* are the elements of the interaction matrix *A*, *s_j_*(*t*) is the expression level of gene *j* at developmental time point *t*, and *s_i_*(*t*+1) is gene *i*’s expression at time *t*+1. In equation 1, *f*(*x*) is a sigmoidal function that determines how the combined regulatory input of all the genes in the network affects the expression of gene *i* (from a value of +1 for complete activation to −1 for complete repression) and is determined by:



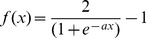
(2)In *f*(x), *a* determines the degree of nonlinearity from repression to activation; in all of the simulations *a* = 1.0, which permitted a range of expression levels and allowed examination of continuous variation in optimal phenotypes in the noise environments.

Gene expression levels were determined by first randomly assigning initial gene expression levels to the **S** vector and then iterating equation 1 over a fixed time period of 100 time steps (analogous to a developmental period). Following previous studies [9], [10], [12], [23], [28], I considered individuals whose gene expression levels achieved a stable state during this developmental time period to be viable and developmentally stable. Genotypes that produced unstable or oscillatory expression patterns were considered unviable. Hence, a mutation to an *A* matrix that shifted a network from a stable expression pattern to one that was oscillatory would be considered a “lethal” mutation. Developmental stability was evaluated after 100 time steps of development by assessing variability in expression levels over the last 10 time steps using:
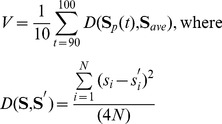
(3)where **S** *_p_*(*t*) was the vector of gene expression values at time *t*, and **S** ***_ave_*** was a vector of gene expression values averaged over the last ten times steps. Following previous studies, if *V* was less than 10^−4^, the phenotype was considered viable [10], [12], [28]. For simplicity, I call this realized gene expression pattern after 100 time steps of development (**S** ***_p_***) the “phenotype” of the individual.

### Dynamics of Selection

At the start of the simulations, populations were seeded with 250 identical, developmentally stable networks. To generate initial networks, *cN* ^2^ ( = 75) entries in an *A* matrix were randomly chosen and randomly assigned a value from a standard normal distribution. Developmental stability was then assessed as described above. This was repeated until a total of 25 developmentally stable, initial networks were generated which were each then used to seed 25 initial populations.

Each of the 25 populations was allowed to evolve for 36000 generations under different forms of selection; responses in robustness, evolvability and epistasis were found to stabilize by after 10000 generations (see Results). Populations were assumed to have non-overlapping generations. At each generation of selection, a pool of 1000 developmentally viable offspring was created by randomly pairing networks within the population and choosing with equal probability rows from each parent’s *A* matrix to form the offspring matrix (as a form of recombination). Inheritance of rows from the parent matrices assumes that cis regulatory elements are tightly linked to genes. Non-zero matrix elements were then allowed to mutate with probability *μ*/(*cN* ^2^) where *μ* = 0.1 (the per genome rate of mutation) with values chosen randomly from a standard normal distribution. Hence, new network connections were not allowed to form; only the strength and direction of regulatory interactions could evolve within a population. Following recombination and mutation, developmental stability of the resultant offspring matrix was assessed as above. A total of 1000 viable offspring matrices were produced with 250 of the most fit representing the next generation. Fitness (*F*) was based on the distance between the offspring phenotype (**S** *_p_*) and the optimal phenotype for the environment (**S** *_opt_*) using the equation:
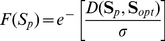
(4)where D is as in equation 3 and *σ* determines the degree of non-linearity of the fitness function. For all simulations, *σ* = 0.05; however, the shape of the fitness function should not influence my results as offspring survival to the next generation was based on fitness rank. **S** ***_opt_*** was dependent on the selection regime the population was experiencing: either stabilizing (no temporal variation), red noise (positively autocorrelated temporal variation), white noise (random temporal variation) or directional selection. For stabilizing selection, **S** *_opt_* was held constant over time and was set equal to the initial phenotype at generation 0 (**S** *_init_*). For red noise environments, reddened time-series were produced using:
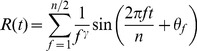
(5)which generates a colored time series by summing sine waves of varying frequency [31], where *n* is the length of the period, *f* is the frequency of each component wave, *t* is the time step (one generation), θ*f* is a uniform random deviate between 0 and 2π that adds random phase to each wave, and γ determines the degree of autocorrelation. For all simulations, I set γ = 1 which produces reddened noise (approximating a sine wave) scaled roughly between −1 and 1 (Fig. S2). The optimal phenotype for the population at generation *t* was then calculated as
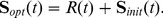
(6)with expression levels allowed to vary up to a maximum of +1 (complete expression) and a minimum of −1 (complete repression). A characteristic of reddened noise is that low frequency fluctuations have larger amplitudes and thus a potentially stronger influence on evolutionary dynamics [32]. In order to evaluate the effects of the periodicity of dominant oscillations, networks experiencing red noise were exposed to four different large amplitude period lengths (n = 10, 30, 50 or 70 generations).

For random, white noise selection regimes, optimal phenotypes changed each generation and were calculated using:
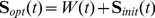
(7)where *W*(t) was a white noise time series and where expression levels were allowed to vary up to a maximum of +1 (complete expression) and a minimum of −1 (complete repression). White noise series were created by randomizing a red noise time series generated using equation 5 and equal in length to the length of the simulation (36000 generations). This ensured that red and white noise regimes experienced comparable levels of variation over time but differed only in their degree of autocorrelation. White noise series were generated using red noise with large amplitude period lengths of *n* = 10, 30, 50 or 70 generations.

Because expression levels were bounded, time-averaged optimal phenotypes in the fluctuating environments could differ from **S** *_init_* and **S** ***_opt_*** in the stabilizing selection environment. To account for potential effects of this variation in time-averaged optima, networks were also exposed to five different directional selection regimes. The optimal phenotypes for the five directional selection environments were chosen from a red noise time series (with a 50 generation, large amplitude period length) at even intervals across half a period of the noise series. Hence, the target optima under directional selection spanned a range of variation comparable to what networks under fluctuating selection experienced.

A potential driver of robustness is recombination load imposed by sexual reproduction [10]. To explore the degree to which robustness, epistasis and evolvability responses were dependent on this mechanism, I performed a more limited numerical analysis of selection with asexually reproducing populations. For these simulations, populations were seeded with the same initial networks as described above, but the offspring pool was produced each generation by randomly choosing and cloning matrices from the parent pool. Networks were allowed to evolve under stabilizing, red noise (period = 50 generations) and white noise (period = 50 generations) selection for 36000 generations.

### Response Variables

Primary response variables were evaluated for 100 randomly chosen networks in each population every 2000 generations from generation 2000 to 36000. To explore evolutionary responses in the early stages of selection, select response variables in the directional, red noise and white noise regimes were also examined every 50 generations from generation 50 to 500. Genetic diversity was measured as the mean number of unique elements per non-zero entry in the A matrices of each population (equivalent to mean allelic diversity). Phenotypic diversity was measured as the number of unique phenotypes within each population (i.e., at least one expression level in the **S** vector was not shared by other individuals in the population). Evolvability, robustness and epistasis were measured at each time point for 100 randomly chosen networks in each population. To measure evolvability of a given network, the network was extracted from its population, cloned and used to seed a new population. This population of 250 identical networks was then exposed to directional selection for four generations towards a randomly generated optimal phenotype (repeated for ten different target phenotypes). Selection proceeded as described above. The rate of adaptation was then measured as the rate at which the ln(Euclidean distance) between the target phenotype and the phenotype of each network in the population decreased per generation using linear regression. Phenotypic variation produced during the evolvability assay was also measured as the standard deviation in Euclidean distances between population phenotypes and the target phenotypes (averaged over the four generations of the evolvability assay). Phenotypic variation in the model is equivalent to additive genetic variation as phenotypic plasticity was not modeled. Evolvability assays were performed using the same mutation rate (*μ* = 0.1) as above. Adaptation rates and phenotypic variation were averaged across targets and networks to obtain mean population-level measures of evolvability.

Robustness and epistatic effects of mutations were assessed by imposing mutations (each replicated 50 times) on randomly chosen non-zero elements of each network (mutant matrix values were chosen randomly from a standard normal distribution). I then examined the relationship between the response variable (*W_m_*) and the number of mutations (*m*), where *W_m_* was either the percent of post-mutation networks that were viable (i.e., developmentally stable) or phenotypic distance (measured as the Euclidean distance between the pre- and post-mutation phenotypes). The choice of the number of mutations to impose was constrained by the fact that maximal effects of mutations were capped in the model at 0% viability at high levels of mutation. Consequently, patterns of epistasis may become asymptotic at high levels of mutation load (resembling antagonistic epistasis), potentially masking patterns of synergistic epistasis at lower levels of mutation accumulation. This is especially problematic if networks that evolve under different selection regimes asymptote at different levels of mutation accumulation, making comparisons among regimes questionable. Accordingly, epistatic effects were explored across a relatively narrow range of one to six mutations. Six mutations was found to reduce average viability to 32% in the initial, pre-selection networks (see Results). This mutation range was also on a scale that was relevant for the evolvability assay in which populations could produce an average of 100 mutations or 0.4 mutations per network per generation.

Mutation effects on viability were modeled using ln(*W_m_*) = −*αm^β^*+ln(*σ*); effects on phenotypic distance were modeled using: *W_m_* = *αm^β^* where *β* measures the strength and direction of epistasis, *α* is the normalization constant, and *σ* = 100 (the maximum percentage of networks that were viable at *m* = 0) [8], [10], [11], [33]. Log-log linear regression was used to estimate *α* and *β* for each network. In these equations, *α* is equivalent to either the log magnitude of the effect of a single mutation on percent viability [*α* = ln(100)−ln(*W_1_*)] or phenotypic distance [*α* = ln(*W_1_*)]. Hence, I used the percent viable under a single mutation (*W_1_*) as a measure of “viability robustness”, calculated as *W_1_* = exp[ln(100)- *α*] or the inverse of phenotypic distance under a single mutation as a measure of “phenotypic robustness”, calculated as 1/*W_1_* = 1/exp(*α*).

For simplicity, results presented in the main text focus on the red noise selection regime with a 50 generation period length (which tended to show the strongest responses) and its corresponding white noise control. Effects of period length on select time-averaged responses can be found in the supporting figures. To assess differences among the initial networks (pre-selection), white noise, stabilizing, directional and red noise selection regimes, response variables were averaged over the last 20000 generations and analyzed using ANOVA. Responses under directional selection were averaged across the five selection targets.

I also examined the relationship between time-averaged evolvability, measured as the rate of adaptation, and several potential predictors including: viability robustness, phenotypic robustness, epistatic effects on viability and epistatic effects on phenotypic distance. Because measures of robustness and epistasis covaried, I used partial least squares regression which accounts for multi-collinearities among independent variables by producing composite latent factors [34]. To interpret loadings of the predictors on the latent factors, I treated predictors with squared weighted loadings greater than 0.05 as statistically significant [34]. All response variables were log transformed to attain homogeneity of variances and normality. Numerical simulations were performed using MATLAB, version 7.10 [35]. All statistical analyses of response variables were performed in R version 2.15 [36].

## Results

While several response variables varied over time, especially under red and white fluctuating selection, linear regressions of response variables versus time revealed no significant trends across selection regimes over the last 20000 generations, indicating that rates of change were on average not significantly different from zero (*P*>0.19, *R* ^2^<0.003, linear regression). The only exception was for log fitness, which exhibited a weak negative relationship with time in the white noise regime (*P = *0.005, *R* ^2^ = 0.025, linear regression).

**Figure 2:**
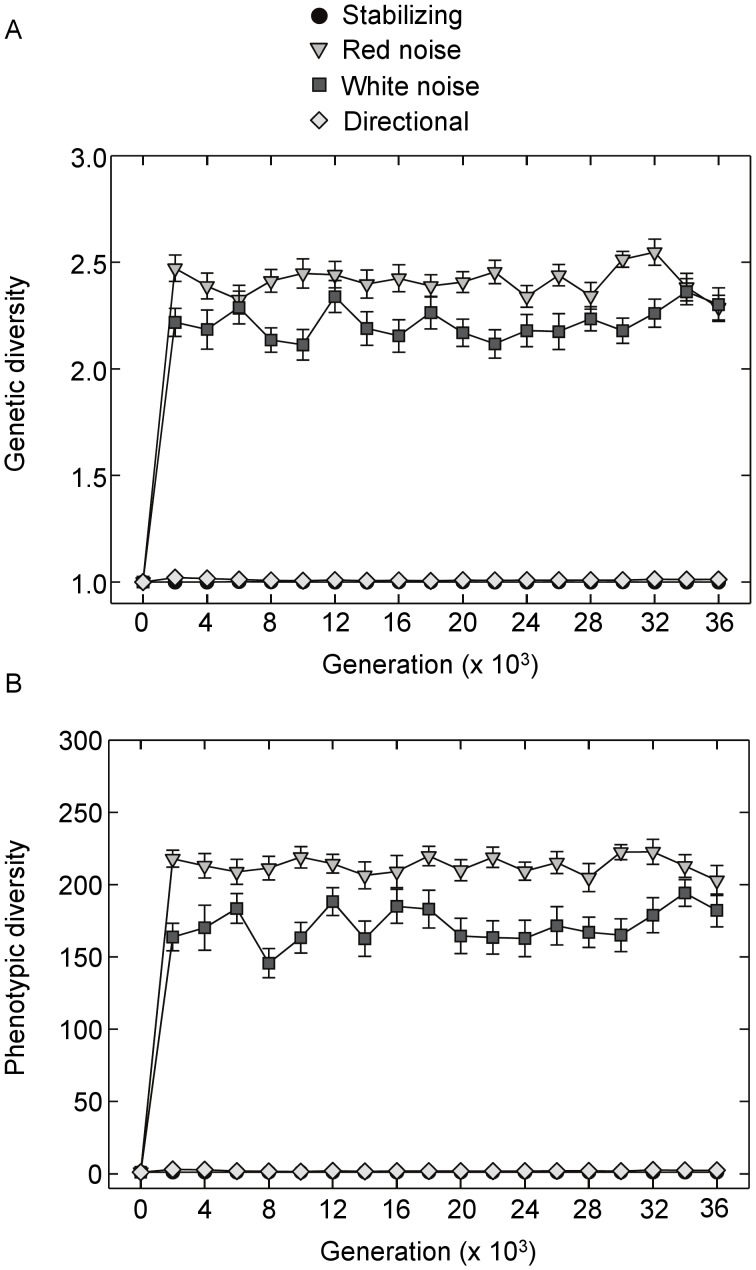
Effects of selection regime on genetic diversity and phenotypic diversity over 36000 generations of selection. (**A**) Genetic diversity (means, +/− S.E.) over time; diversity of each population was measured as the mean number of alleles per locus across all A matrices. (**B**) Phenotypic diversity (means, +/− S.E.) measured as the number of unique phenotypes within each population. Results for the fluctuating environments are for red noise with a 50 generation period length and its corresponding white noise control.

Temporal heterogeneity (both random and autocorrelated) resulted in rapid accumulation of genetic and phenotypic diversity within populations (Fig. 2). Time-averaged phenotypic and genetic diversity varied significantly among selection regimes (Fig. 2; *P*<0.001, ANOVA). Both measures were higher in the red and white noise environments compared to the initial networks and the stabilizing and directional selection regimes; diversity was also higher in the red noise environment compared to white noise (all *P*<0.001, Tukey’s HSD test). No differences were observed among the directional, initial and stabilizing networks (*P*>0.80, Tukey’s HSD).

**Figure 3:**
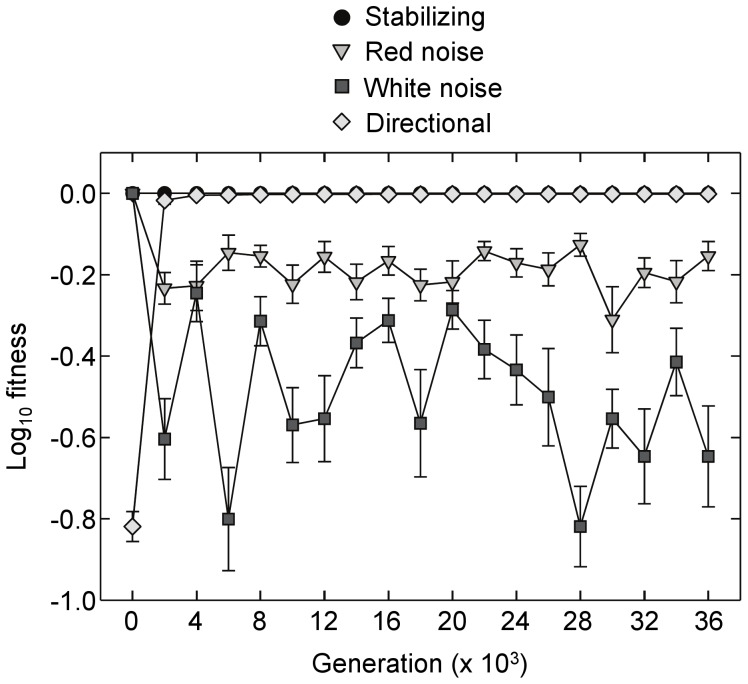
Log fitness over time. Shown are means across populations (+/−SE) over 36000 generations of selection in the four selection regimes. Results for the fluctuating environments are for red noise with a 50 generation period length and its corresponding white noise control.

Mean fitness averaged across populations also stabilized relatively rapidly, though fluctuations were apparent over time (Fig. 3). Time-averaged log fitness (i.e. geometric mean fitness) varied significantly among selection regimes (*F*_ 4,120_ = 459.2, *P*<0.001, ANOVA) with levels significantly lower in the fluctuating environments compared to the initial, stabilizing and directional selection networks (*P*<0.001, Tukey’s HSD test). Geometric mean fitness was also higher in the red noise environment compared to populations that experienced white noise (*P*<0.001, Tukey’s HSD test).

**Figure 4:**
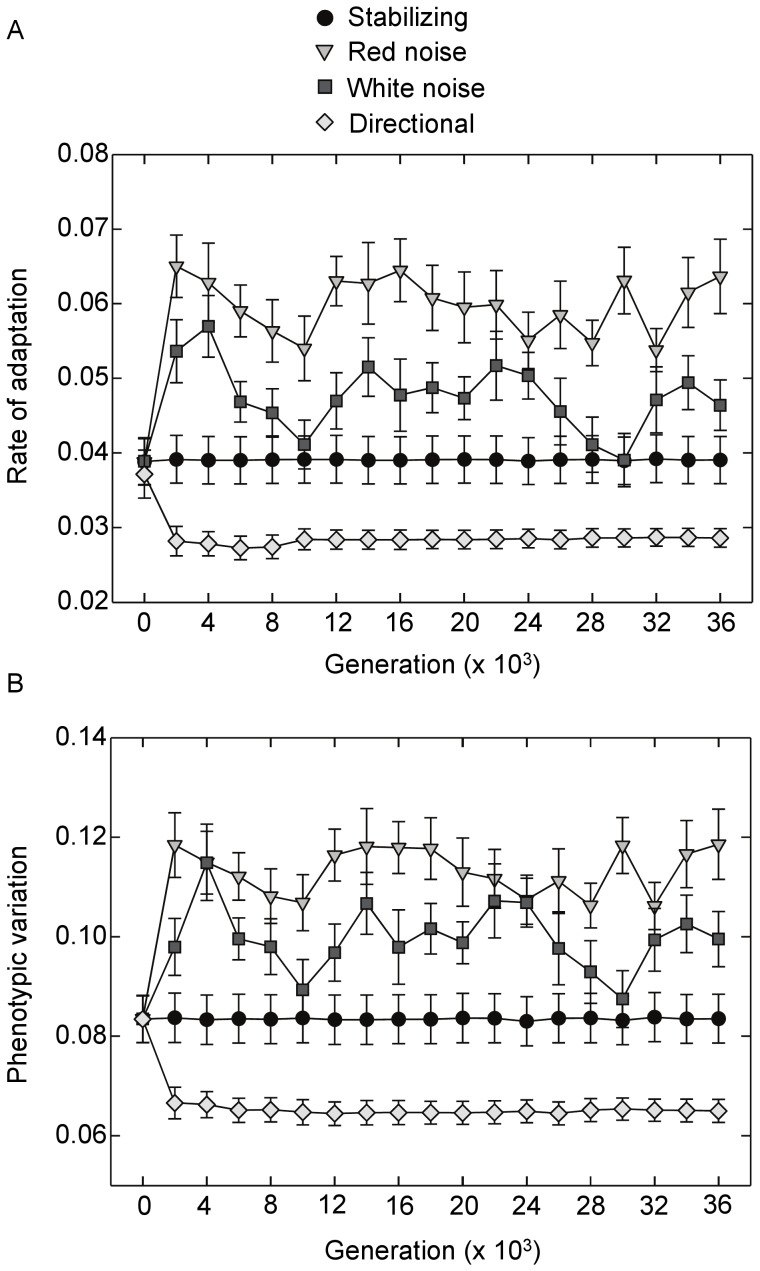
Effects of selection on evolvability over time. Evolvability was measured as the rate of adaptation and the production of phenotypic variation assessed under selection in novel environments. Measures were determined using 100 networks randomly selected from each population every 2000 generations. (**A**) Mean evolvability (+/− SE) over time. (**B**) Mean production of phenotypic variation (+/− SE) over time. Results for the fluctuating environments are for red noise with a 50 generation period length and its corresponding white noise control.

Evolvability measured as either the rate of adaptation or production of phenotypic variation from the evolvability assays varied significantly among source environments (*P*<0.001, ANOVA). Only networks that evolved under red noise exhibited significant increases in their time-averaged measures of evolvability relative to the initial networks (Fig. 4A, 4B; rate of adaptation: *P* = 0.003, Tukey’s HSD; phenotypic variation: *P* = 0.004, Tukey’s HSD). Evolvability showed the opposite pattern under directional selection, decreasing relative to mean initial levels (rate of adaptation: *P* = 0.023, Tukey’s HSD; phenotypic variation: *P* = 0.015, Tukey’s HSD). There was some evidence that evolvability increased in the white noise environments as well (Fig. 4A, 4B). Indeed, no significant difference between red and white noise environments was detected for either production of phenotypic variation (*P* = 0.46, Tukey’s HSD test) or the rate of adaptation (*P* = 0.20, Tukey’s HSD test). However, mean evolvability in the white noise environment was also not significantly different from initial levels, indicating that selection for evolvability was weaker compared to red noise environments (rate of adaptation: *P* = 0.51, Tukey’s HSD test; phenotypic variation: *P* = 0.46, Tukey’s HSD). As expected, there was a strong positive relationship between the generation of phenotypic variation and the rate of adaptation in the evolvability assay (*P*<0.001, *R^2^* = 0.92, linear regression between time-averages).

A potential confounding factor in the evolvability assays was the initial difference between the randomly generated target phenotypes and the phenotypes of the networks being assayed. As there is likely a limit to how closely a network can match a target optimal phenotype when under directional selection, networks that are initially very similar to their targets may exhibit little or no adaptive change over time. To test and control for this, I analyzed evolvability measured as the mean rate of adaptation using ANCOVA in which initial Euclidean distance from the target was used as the continuous covariate and the fixed effect was environment (either initial, stabilizing, directional, red noise or white noise). Evolvability was positively related to mean initial distance (*F*_1,119_ = 10.9, *P*<0.001, ANCOVA), though effects of initial distance were driven by two outlier values from a single initial population with very low initial distance and low evolvability (Fig. S3). The interaction between the covariate and environment was not significant (*P* = 0.49, ANCOVA). However, effects of selection regime were still significant after controlling for the covariate (*F*_ 4,119_ = 27.1, *P*<0.001, ANCOVA).

**Figure 5:**
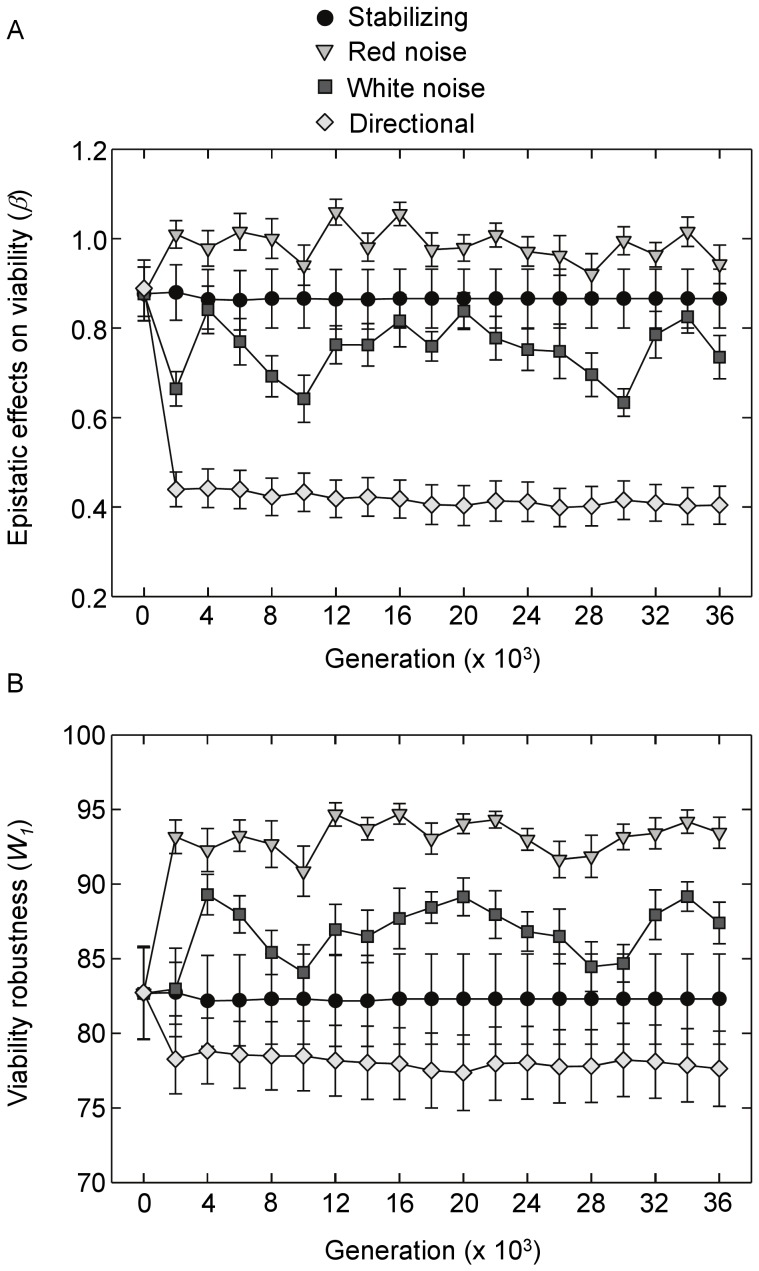
Epistatic effects of mutations on viability and viability robustness over time. Shown are mean responses (+/−SE) under the four selection regimes. (**A**) Epistatic effects of mutations (*β*) over time, measured as effects on the percentage of post-mutation phenotypes that were viable. (**B**) Viability robustness (*W_1_*) over time measured as the percentage of networks that were viable under one mutation, *W_1_* = exp[ln(100)-*α*]. Results for the fluctuating environments are for red noise with a 50 generation period length and its corresponding white noise control.

When examining epistatic effects of mutations on network viability, significant effects of selection on time-averages were present (Fig. 5A; *F*_ 4,120_ = 36.5, *P*<0.001, ANOVA of time averages). Stabilizing, red noise, and white noise selection did not affect mean viability epistasis relative to initial levels (all *P*>0.63, Tukey’s HSD test). However, time-averaged b values were lower under directional selection relative to initial levels (*P*<0.001, Tukey’s HSD test). Effects of selection on viability robustness were also evident (Fig. 5B; *F*_ 4,120_ = 7.8, *P*<0.001, ANOVA of time averages). Networks that evolved under red noise were on average more robust compared to initial levels (*P* = 0.024, Tukey’s HSD test). In contrast, stabilizing and white noise selection did not differ from mean initial values (*P*>0.54, Tukey’s HSD test), while viability robustness declined under directional selection relative to initial levels (*P* = 0.03, Tukey’s HSD test).

**Figure 6:**
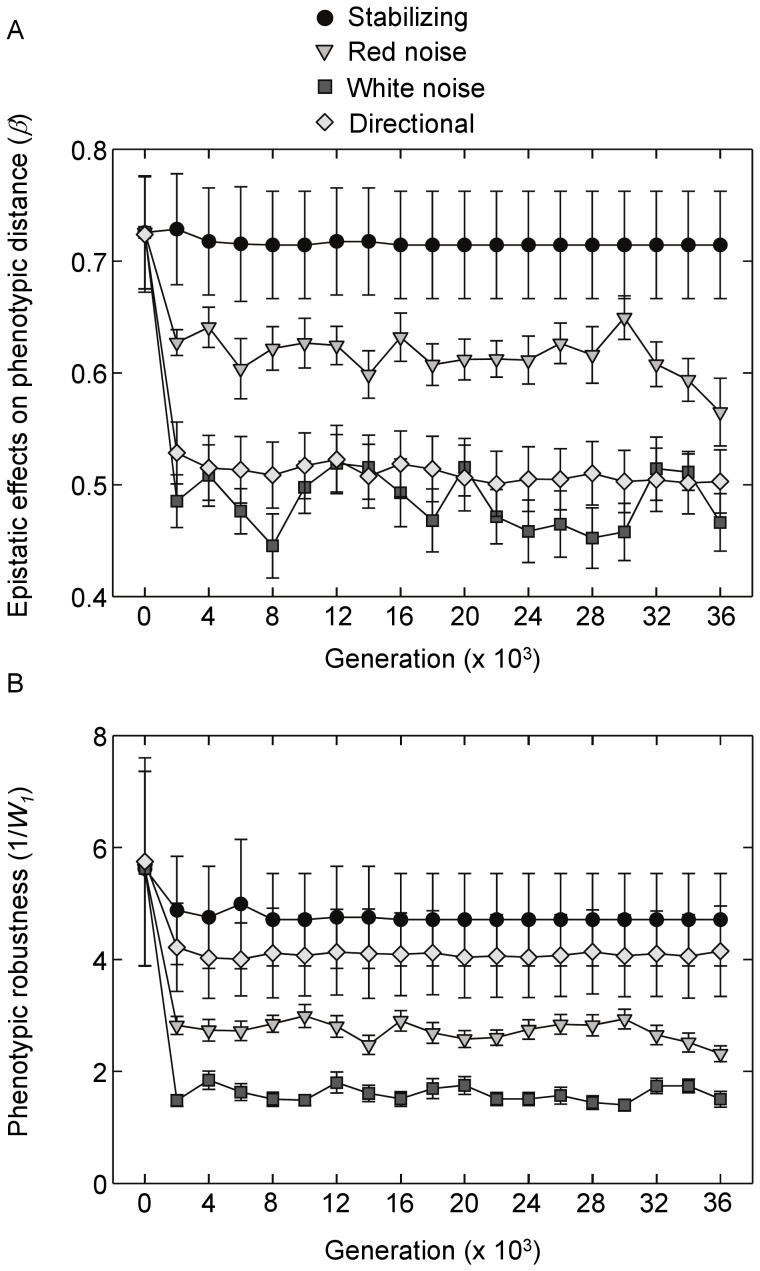
Epistatic effects of mutations on phenotypic distance and phenotypic robustness over time. Shown are mean responses (+/−SE) under the four selection regimes. (**A**) Epistatic effects on phenotypic distance (*β*) over time, measured as the Euclidean distance between post- and pre-mutation phenotypes. (**B**) Phenotypic robustness (1/*W_1_*) over time measured as the inverse of phenotypic distance under a single mutation, 1/*W_1_* = 1/exp(*α*). Results for the fluctuating environments are for red noise with a 50 generation period length and its corresponding white noise control.

Epistatic effects on phenotypic distance also varied among selection regimes (Fig. 6A; *F*_4,120_ = 35.7, *P*<0.001, ANOVA of time averages). Stabilizing and red noise selection did not affect mean phenotypic epistasis relative to initial levels (all *P*>0.10, Tukey’s HSD test). However, time-averaged *β* values were lower under both directional and white noise selection relative to initial levels (*P*<0.001, Tukey’s HSD test). *P*henotypic robustness also responded to selection (Fig. 6B; *F*_ 4,120_ = 18.3, *P*<0.001, ANOVA of time averages). Networks that evolved under red noise, white noise and directional selection were on average less robust compared to initial levels (*P*<0.047, Tukey’s HSD test); stabilizing selection did not differ from initial levels (*P*>0.99, Tukey’s HSD test).

The patterns revealed in Figures 5 and 6 suggest that epistatic effects (*β*) covary negatively with average effects of mutations (*α*). A nonlinear, concave relationship between time-averaged values of *β* and *α* was evident for both phenotypic distance and viability (Fig. S4). Mean fitness profiles based on *β* and *α* for networks that evolved under the four primary selection regimes and for the initial, pre-selection networks are displayed in Figure S5. A potential concern is that the range of mutations used to estimate *β* and *α* may have been inadequate to capture true patterns of robustness and epistasis. As described in the Methods, the small range of mutations employed was done to avoid masking relationships at the low end of the mutation gradient with relationships that may become asymptotic at high levels of mutation load. However, further analyses using up to 50 mutations showed that relationships, while quantitatively different, were qualitatively indistinguishable from the above results at generation 36000 (Fig. S6).

**Figure 7:**
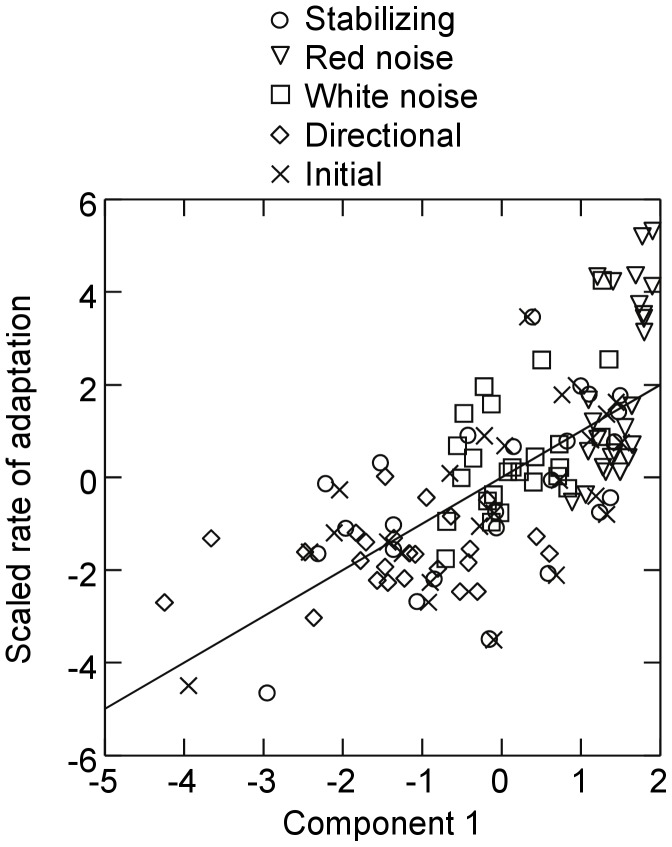
Results of partial least squares regression examining the relationship between evolvability (the rate of adaptation) and measures of epistasis and robustness. Shown are results for the first component axis of the regression which accounted for 40.3% of variation in evolvability. With the exception of the initial pre-selection networks, data were based on time averages over the last 20000 generations. Loadings of explanatory variables on the component axis can be found in Table 1. Results for the fluctuating environments are for red noise with a 50 generation period length and its corresponding white noise control.

Partial least squares regression of mean population evolvability, measured as the rate of adaptation, versus potential predictors generated a first component axis that accounted for 40.3% of variation in evolvability and 43.6% of variation in the predictor variables (Fig. 7). Note that the dependent variable has been rescaled by subtracting values from the grand mean and dividing by its standard deviation – a standard procedure in this statistical test. Both viability robustness and epistatic effects on viability loaded strongly and positively with the first component axis followed by decreasing phenotypic robustness (Table 1). Hence, increasing evolvability across all populations and source environments increased with increasing population-level viability robustness and viability epistasis and decreasing phenotypic robustness. Phenotypic robustness and epistatic effects on phenotypic distance loaded most strongly and negatively on the second component axis, accounting for an additional 43.4% variation in the predictor variables. However, the second component had very little explanatory power, only accounting for an additional 0.6% variation in evolvability. The coupling of viability robustness and evolvability was also evident in the time series; oscillations in evolvability over time in the red and white noise environments were paralleled by oscillations in viability robustness (Fig. 4 versus Fig. 5B). Cross-correlation analysis revealed a positive correlation between the two measures in both the white noise (*R* = 0.49, *P*<0.0001, time lag = 0) and red noise environments (*R* = 0.45, *P*<0.0001, time lag = 0).

**Table 1:** Loadings of predictor variables on the first two components produced by the partial least squares regression analysis of evolvability.

Predictor	Component 1	Component 2
Phenotypic epistasis	−0.074	−0.698*
Viability epistasis	0.659*	−0.309
Phenotypic robustness	−0.347*	−0.703*
Viability robustness	0.673*	−0.31*

Predictors with squared weighted loadings >0.05 were treated as statistically significant (indicated by *).

The dominant period length of red noise had no effects on genetic diversity in either red or white noise environments (Fig. S7). However, period length influenced phenotypic diversity. In the white noise environments, no effects were detected; in the red noise environments, phenotypic diversity increased with period length, peaking at the 50 generation period (Fig. S7). In both red and white noise environments, there were no effects of dominant period length of red noise on evolvability measured as the capacity to produce phenotypic variation (Fig. S8). However, a significant effect of period length was detected when measuring evolvability as the rate of adaptation (Fig. S8); evolvability under the 70 generation period length was significantly lower compared to the 50 generation period length. Significant effects of period length were also observed for both viability robustness and epistatic effects on viability (Fig. S9). For both measures, no effects of period length were observed under white noise (*P*>0.90, Tukey’s HSD test). However, under red noise, levels of robustness and epistasis were higher in the 50 generation period environment compared to all other period lengths (*P*<0.001, Tukey’s HSD test; *P*>0.10 all other comparisons, Tukey’s HSD test). As with measures of mutation effects on viability, effects on phenotypic distance (both robustness and epistasis) were not affected by period length in the white noise environments (Fig. S10). In contrast, both measures were affected by red noise, increasing in magnitude with period length.

Reproductive mode significantly altered the effects of selection on key response variables; responses were weaker under asexual reproduction compared to sexual reproduction (Fig. S11). I used two-way ANOVA to examine the effects of reproductive mode and source environment (stabilizing, red noise, and white noise selection and initial networks) on responses averaged over the last 20000 generations of selection. When analyzing evolvability measured as the rate of adaptation, responses were clearly weaker in fluctuating environments with asexual reproduction. A weak interaction between reproductive mode and environment was detected (Fig. S11; *F*_3,192_ = 2.6, *P* = 0.06, ANOVA). While a trend for enhanced evolvability in the asexual red noise environment was present, pairwise comparisons among asexual populations revealed no differences between selection regimes and initial levels (*P*>0.50, Tukey’s HSD test). Viability robustness was also lower in asexual populations compared to sexual populations that experienced fluctuating selection (Fig. S11). Reproductive mode interacted with source environment when analyzing viability robustness (*F*_3,192_ = 2.8, *P* = 0.04, ANOVA); among asexual populations, no effects of selection were detected (*P*>0.50, Tukey’s HSD test). Similarly, reproductive mode and selection interactively affected geometric mean fitness (time-averaged log fitness); fitness was lower in the fluctuating environments with asexual reproduction compared to sexual populations (Fig. S11). Among the asexual populations, fitness in the red and white noise environments was lower than initial levels (*P*<0.001, Tukey’s HSD test) but was significantly higher under red noise compared to white noise environments (*P*<0.001, Tukey’s HSD test).

## Discussion

My results show that evolvability and the capacity to generate phenotypic variation can emerge when selective forces fluctuate and that such effects are stronger when fluctuations are temporally autocorrelated. A key characteristic of reddened noise is that autocorrelated low frequency fluctuations have larger amplitudes and thus a potentially stronger influence on evolutionary dynamics. Reddened noise whose large amplitude oscillations occur at a high frequency (i.e. small period length) may provide little opportunity for populations to evolutionarily track changing environmental conditions, reducing the potential for the accumulation of genetic diversity and the evolution of emergent network properties such as enhanced evolvability and robustness. Conversely, extremely long period lengths whose rate of major environmental change is slow should reduce the selective advantages of rapid adaptive capacity and robustness. Hence, population genetic and network properties may be expected to show a unimodal relationship with period length in reddened environments. My results provided some support for this general prediction. Phenotypic diversity and mutational effects on viability and phenotypic distance increased with increasing period length and tended to peak at the intermediate 50 generation period length. Despite this, effects on measures of evolvability were weak; while evolvability displayed a trend for greater values in the 50 generation period length environment, effects were not statistically strong.

Clearly the ability of a genetic system to generate heritable phenotypic variation and adapt to a changing environment will be intimately related to the manner in which mutations affect gene expression. However, such responses are multi-faceted, being a function of the system’s inherent robustness to mutations as well as the manner in which effects of accumulating mutations are dependent on the genetic background. The emergence of greater evolvability under red noise was paralleled by an increase in viability robustness. When examining potential drivers of evolvability across all environments, populations that on average exhibited greater viability robustness and epistatic responses in viability to mutations exhibited greater evolvability. This coupling was also apparent in the dynamics of evolvability under fluctuating selection. Evolvability in the red and white noise environments oscillated over time and was mirrored by oscillations in viability robustness. The manner by which enhanced robustness to lethal mutations can increase evolvability is fairly intuitive. Enhancing a network’s capacity to maintain developmental stability in the face of accumulating mutations ensures that a greater proportion of mutant phenotypes will be viable and present in the next generation’s offspring pool. Thus, there is a greater probability that mutations will result in phenotypic variation in subsequent generations. This may permit genomes to incrementally explore genotype/phenotype space [4], [29] - a characteristic that should enhance adaptive responses to changing environmental conditions especially under red shifted noise where fluctuations are autocorrelated and phenotypic optima are more similar for points closer in time.

An interesting outcome was that directional selection resulted in a decrease in viability robustness and evolvability over time. This is clearer when examining more detailed evolutionary dynamics near the beginning of the simulations (Fig. S12). This shows clearly an initial increase in population genetic diversity under directional selection which was mirrored by an initial increase in viability robustness and evolvability. However, as populations began to approach their optimal phenotypes (as shown by increasing fitness), genetic diversity declined as did viability robustness and evolvability. One possible explanation is that reduced viability robustness allows populations to more efficiently purge deleterious mutations via purifying selection and reduce the prevalence of non-optimal phenotypes - a feature that should be favored when populations are at or nearing their optimal phenotypes and under increasing stabilizing selection. This general mechanism has been invoked to explain the evolution of directional epistasis in sexual populations and has been shown to operate in prior analyses of this model system [10], [37].

In addition to mutation effects on viability, I also examined effects on phenotypic distance. It is an intuitively appealing idea that genotypes that produce offspring that differ greatly from parent phenotypes could adapt more rapidly to changing environments. Thus, an increase in mutation effects on phenotypic distance (reduced phenotypic robustness and increased epistatic effects) was predicted to lead to greater evolvability. Partial least squares regressions provided some support for this prediction: evolvability showed a positive relationship with the first component axis which was negatively associated with phenotypic robustness. Phenotypic robustness also responded negatively to selection in the red and white noise environments while epistatic effects on phenotypes increased under white noise. Despite this, phenotypic robustness did not appear to be a primary driver of evolvability. For instance, in the partial least squares regression analysis, phenotypic robustness loaded weakly on the first predictor axis when compared to measures of viability robustness and epistasis. Instead, mutation effects on phenotypic distance loaded most strongly on the second predictor axis which explained a minor proportion of variation in evolvability among populations. Furthermore, when compared to responses under white noise, phenotypic robustness responded weakly in the red noise environment, where evolvability responded the most strongly. A potential explanation is apparent when comparing patterns of viability robustness versus phenotypic robustness - the two appeared to covary positively. A significant correlation between time averages of the two measures was present when analyzing across all populations and environments (*R* = 0.34, *P*<0.0001, Pearson correlation). Time series analysis also revealed significant, positive cross correlations between phenotypic robustness and viability robustness for the red noise (*R* = 0.28, *P*<0.001, time lag = 0) and white noise environments (*R* = 0.29, *P*<0.001, time lag = 0). Thus, the capacity to generate highly variant offspring phenotypes (low phenotypic robustness) may be evolutionarily constrained by increases in the lethal effects of mutations and reduced offspring viability (low viability robustness). This may also explain why epistatic effects on phenotypic distance increased under directional selection, where such effects would appear to be non-adaptive once optimal phenotypes have been attained. Selection for reduced viability robustness under directional selection, as a means to enhance purifying selection, may have resulted in correlated selection for increased mutation effects on phenotypic distance.

Whereas increases in viability robustness and, to a lesser extent, decreases in phenotypic robustness accounted for differences in evolvability, partial least squares regression revealed that evolvability was also associated with increasing epistatic costs of mutations on viability. Similarly, epistatic effects on phenotypic distance declined in white noise environments where phenotypic robustness was lowest. These seemingly paradoxical results can be reconciled by considering the nonlinear trade-off between *β* and *α* (Fig. S4). For example, in the case of mutation effects on viability, costs due to increasing epistasis (increasing *β*) are small relative to fitness gains due to increasing robustness and decreasing *α* across a broad range of *α* values. The consequences of this becomes apparent when examining mean fitness profiles for networks that evolved in red noise environments (where selection for increased viability epistasis was highest) versus white noise, stabilizing and initial networks (Fig. S5). Even though the effects of deleterious mutations in red noise networks become more harmful as they accumulate, the overall fitness costs of mutations are much smaller compared to white noise and stabilizing networks over a range of mutation accumulation. As an example, red noise networks with four mutations maintain 76% viability on average. In contrast, viability drops to 49% for stabilizing selection networks with the same mutation load. Prior models of genetic interactions have predicted that robustness and the strength of epistatic effects should covary negatively [8]. My results support this contention but also highlight the importance of knowing the shape of the trade-off for comprehending the fitness costs and benefits of changes in *α* and *β*.

That epistasis and viability robustness can evolve is consistent with prior examinations that have utilized this model framework [10], [12], [13]. One study using this model system has also demonstrated that evolvability can emerge under fluctuating selection [23], though the autocorrelational structure of the temporal variation was not described. This study also showed that increases in the capacity to evolve were correlated with an increase in the capacity of networks to generate phenotypic change under mutation, interpreted as a decrease in robustness. This is similar to my finding that robustness in phenotypic expression levels decreased under fluctuating selection and was associated with increases in evolvability. As effects on network viability were not addressed in [23], it is not known if decreases in phenotypic robustness were accompanied by increases in viability robustness or if such effects were correlated with changes in the strength and direction of epistasis. A number of studies using this model system have also shown that selection for stable gene expression patterns (viability selection) can impose selection for genetic robustness in the presence of stabilizing selection or even in the absence of external selective forces [10], [12], [13]. It is interesting then that viability robustness did not increase significantly under stabilizing selection in my analysis. A possible explanation is that prior studies have used arbitrary fitness cutoffs to determine inclusion of offspring in subsequent generations as offspring were produced via random reproduction. This may increase variation in offspring fitness and genetic composition which could drive selection for viability robustness. In my study, a large offspring pool was first generated and only the most fit networks were then allowed to populate the next generation, reducing the possibility that suboptimal phenotypes and genetic variation were introduced into the subsequent generation. Because populations started with all individuals at their optimal phenotypes under stabilizing selection, little room was available for the introduction of genetic diversity. It is important to note that, despite this, gene networks did evolve over time under stabilizing selection. Figure S13 displays time series of the proportion of network regulatory elements (*a*_ij_ elements of the *A* matrix) that changed relative to their initial values for the different selection regimes. As expected, the majority of regulatory elements rapidly changed under directional and fluctuating selection. However, networks under stabilizing selection also exhibited a smaller but significant degree of change, indicative of a slow accumulation of neutral or near-neutral mutations over time.

Discussion of the effects of population genetic variation and viability selection as evolutionary drivers of robustness naturally leads to the question of whether evolvability was under direct selection in fluctuating environments or simply a correlated response to selection for robustness under enhanced genetic diversity. The importance of temporal heterogeneity in the maintenance of genetic variation remains controversial [38], [39]; my study supports previous findings that temporally varying selection can, in theory, promote genetic and phenotypic diversity within populations [15], [16], [38], [40]. High standing genetic/phenotypic variation can promote rapid responses to changing environmental conditions and confer high population-level evolvability. Thus, higher fitness observed in the red noise environments relative to white noise could be partially due to differences in genetic variation in addition to differences in individual, network-level evolvability and robustness. Additionally, high levels of genetic diversity could have imposed a recombination load on populations as reproduction reshuffled genotypes and produced unfavorable genetic combinations. This, in turn, may have selected for increased robustness and indirect selection for evolvability. As described above, prior studies have found that selection for stable gene expression patterns in the absence of external selection can itself select for robustness as populations accumulate genetic variation via mutation [10], [12], [13]. This mechanism was apparent in the early stages of directional selection where an initial increase in genetic diversity was correlated with an increase in viability robustness and evolvability that diminished over time as populations approached their evolutionary optima (Fig. S11). While this effect was minimized by the stabilizing selection regime I implemented (see above), fluctuating selection (both red and white noise) allowed the introduction and maintenance of genetic diversity, which may have contributed to selection for robustness via selection for developmental stability. To examine this further, I performed a supplemental numerical analysis to assess the effects of recombination load, in which populations reproduced asexually. While asexual populations in reddened environments maintained significantly higher fitness relative to the white noise environment, effects of red noise on robustness and evolvability in the asexual populations were weaker compared to sexual populations (Fig. S10). Although, asexual populations showed a trend for higher evolvability in the red noise environments, effects on both viability robustness and evolvability were not statistically significant. Hence, this cursory analysis indicates that genetic variation and recombination load may have combined to select for robustness and evolvability.

The appearance of an adaptive response in robustness and evolvability naturally leads to the question of an underlying mechanism. In my analysis, patterns of regulatory connections were held constant so evolutionary changes in topology cannot account for my findings [30]; only the strength and direction of genetic interactions could respond to selection. To analyze changes in the strength and sign of interactions at the end of the simulations, inter-gene regulatory interactions (off-diagonal *a*_ij_ elements of the *A* matrix) were first multiplied by expression levels to determine the sign of the interaction. This was necessary since the sign of the effect of a gene on another gene was dependent not only on *A* matrix elements but on expression levels which themselves varied between −1 and 1, affecting the direction of regulatory interactions. Absolute values were used as measures of interaction strength. This analysis revealed a significant increase in interaction strength under directional selection relative to initial levels. Also evident was a significant shift to a greater proportion of positive regulatory interactions in the red noise environments (Fig. S14). When analyzing across all environments, pairwise correlations of time averages revealed a significant positive relationship between the proportion of positive regulatory interactions and both evolvability (Fig. S15; *R* = 0.42, *P*<0.001) and viability robustness (Fig. S15; *R* = 0.28, *P* = 0.016). A greater number of positive interactions should enhance robustness of genes that are at or near full expression. This is clearer when referring back to equations 1 and 2 which govern gene expression. For each gene, expression is a nonlinear, sigmoidal function of regulatory inputs from other genes in the network. A greater number of positive interactions increases the likelihood that expression levels are in the saturating range of the expression function where changes in regulatory inputs via mutations will have relatively small effects on realized expression. Since changes in expression levels cascade to other genes via regulatory interactions, any mechanism that maintains expression under mutation should dampen such cascading effects and help maintain developmental stability.

While my results provide evidence that greater robustness and evolvability can emerge under autocorrelated environmental fluctuations, some caveats are warranted. First, for such emergent properties to evolve through direct selection, polymorphism for the property must be present within populations, the likelihood of which is greater with higher mutation rates and larger population sizes [7], [41]. The probability of polymorphism increases and selection for robustness becomes more effective when N*µ*_n_>1/n, where N is the effective population size, *µ*_n_ is the mutation rate per gene and n is the number of genes in the network [7]. For my model, I employed a relatively high mutation rate of *µ*_n_ = 0.0013 per gene per generation. Thus, N*µ*_n_ = (250)(0.0013) = 0.325, which was greater than 1/n = 0.013, increasing the potential for strong selection for robustness. Supplemental simulations using a mutation rate of *µ*_n_ = 0.000013 (N*µ*_n_ = 0.00325) confirmed that robustness and evolvability failed to evolve in red noise environments at these lower mutation rates (data not shown). While reported mutation rates are much lower in nature compared to those used here - on the order of 10^−10^ per gene per replication [42] - larger population sizes could compensate for lower mutation rates and allow for polymorphism and selection for robustness in natural systems. This would be especially true for microbial organisms which can attain extremely large population sizes.

### Conclusions

As with any model, the system presented here is a highly simplistic abstraction of exceedingly complex phenomena. Computational resources place limitations on the scope of numerical models, limiting in the present case the size of populations, the number of replicates and the size of the regulatory networks analyzed. Whether my results scale up to much more complex, natural settings is an open question. For instance, it was assumed that all genes had the potential to interact, increasing the possibility of epistatic interactions. Employing a model framework in which some genes act as modifiers (mediating regulatory interactions) and others act independently to influence phenotypes could produce divergent results. I also did not allow network connectance to respond to selection, setting the number of regulatory connections at a constant value, comparable to previous studies that used this model system [10], [12]. Allowing the number of connections to evolve was outside the scope of the present study but could provide valuable insight into how networks respond to fluctuating environmental conditions. My work also only considered mutational robustness and not the capacity of networks to buffer the impacts of environmental noise on development and gene expression (an important facet of canalization in natural systems). Missing too is phenotypic plasticity - a potentially important mediator of responses to fluctuating environmental conditions. Mutational robustness in living systems can also involve several mechanisms not encompassed in the present model such as proofreading during replication. Moreover, the mapping of the genotype to phenotype is especially multi-layered for metazoans in which epigenetic phenomena and development offer a hierarchy of processes that determines phenotypic expression and mediates robustness [43], [44]. Applicability and empirical tests of the model presented here may be more appropriate for simpler unicellular taxa, especially haploid organisms such as bacteria. These taxa can attain the large population sizes and high mutation rates required to generate polymorphism and effective selection for robustness and evolvability. Moreover, evidence of evolutionary changes in metabolic network structure in response to variable environments is known for a variety of bacteria species [45].

Recent debate has arisen concerning the role of adaptation versus neutral processes in the generation of complexity and emergent properties of genetic systems [46], [47]. My results support the view that robustness and evolvability are traits that can emerge under fluctuating selection. This finding consolidates several recent model investigations that show that changing phenotypic optima can lead to the emergence of robustness and/or enhanced adaptability in evolving gene regulatory networks or protein networks [4], [23], [48]–[50]. My results add to this foundation by highlighting several important features that are likely to be of great importance in natural systems. First, the degree of autocorrelation in the environmental noise that populations experience can be a vital determinant of whether robustness and evolvability emerge as adaptations to variable conditions. Secondly, reddened noise may select for evolvability indirectly by maintaining genetic variation which in turn selects for robustness to reduce recombination load and maintain developmental stability. This effect may combine with selection for evolvability in a positive feedback loop that catalyzes the evolution of these emergent properties. In short, red noise selects for robust genotypes that promote population-level genetic variation which in turn selects for enhanced robustness to minimize recombination load and maximize developmental stability which further enhances evolvability. Finally, my model demonstrates that organisms may face trade-offs during selection for reduction in the negative impacts of mutations as optimization of either genetic robustness or epistatic effects comes at the cost of the other. At present, little empirical evidence exists to assess whether robustness and epistatic effects covary negatively and if so, what the shape of the trade-off curve is. Predicting which traits will be at a greater selective advantage in natural systems and in response to fluctuating environments may depend vitally on exposing such relationships.

## Supporting Information

Figure S1 **Effects of selection on evolvability (the rate of adaptation) for networks with 0.20 and 0.80 connectance (c).** Results were measured after 2000 generations of selection on 25 randomly generated initial networks. Results for the fluctuating environments are for red noise with a 50 generation period length and its corresponding white noise control. Shown are means +/−S.E.
(TIF)

Figure S2 **An example of a red noise time series.** Results were generated using equation 4, with a period length of the largest amplitude fluctuation equal to 20 generations, a time step of one generation, and g = 1.
(TIF)

Figure S3 **The relationship between the initial distance to evolvability target phenotypes and evolvability measured as the rate of adaptation.** Results are population means (+/− S.E.). Shown is the linear regression fit.
(TIF)

Figure S4 **The relationship between epistatic effects (** ***β*** **) and the normalization constant (** ***α*** **).** **(A)** Results based on mutation effects on log percent viability. **(B)** Results based on mutation effects on phenotypic distance. Shown is the fit for the power relationship b = −aα^z^+σ. Data are from the initial networks and time-averages over the last 20000 generations of selection for four selection regimes. Results for the fluctuating environments are for red noise with a 50 generation period length and its corresponding white noise control. Symbols as in Fig. S3.
(TIF)

Figure S5 **Mutation effects on components of fitness.** Separate relationships are shown for the initial (pre-selection) networks and networks that experienced selection under stabilizing, directional, red noise (period = 50 generations) and white noise (period = 50 generations) selection. **(A)** The relationship between the number of mutations (*m*) and the natural log of the percentage of networks that are viable (*W_m_*). Curves were generated using the relationship ln(*W_m_*) = −*αm^β^*+ ln(100). **(B)** The relationship between the number of mutations (*m*) and the phenotypic distance between mutant and pre-mutation phenotypes (*W_m_*). Curves were generated using the relationship *W_m_* = *αm^β^*. In the case of networks under selection, *α* and *β* were first estimated as time-averages from model results over the last 20000 generations then averaged across populations.
(TIF)

Figure S6 **Effects of selection on epistasis and robustness measured using an expanded range of mutations (1–50).** Shown are results for the initial (pre-selection) networks and networks after 36000 generations under stabilizing, directional, red noise (period = 50 generations) and white noise (period = 50 generations) selection (means, +/−S.E.). **(A)** Effects on viability robustness (*W_1_*). A significant effect of environment was detected (*F*_3,96_ = 8.4, *P*<0.001, ANOVA); robustness of red and white noise networks was significantly greater than initial levels (*P*<0.001, Tukey’s HSD test). **(B)** Effects on epistatic effects on viability (*β*). A significant effect of environment was detected (*F*_3,96_ = 7.1, *P*<0.001, ANOVA); *β* of red noise networks was significantly greater than initial levels (*P*<0.001, Tukey’s HSD test; *P* = 0.84 for white noise versus initial). **(C)** Effects on phenotypic robustness (1/*W_1_*). A significant effect of environment was detected (*F*_3,96_ = 6.6, *P*<0.001, ANOVA); robustness of red and white noise networks was significantly lower than initial levels (*P*<0.02, Tukey’s HSD test). **(D)** Effects on epistatic effects on phenotypic distance (*β*). A significant effect of environment was detected (*F*_3,96_ = 21.3, *P*<0.001, ANOVA); *β* in red and white noise networks was significantly lower than initial levels (*P*<0.01, Tukey’s HSD test).
(TIF)

Figure S7 **Effect of the dominant period length of red noise on genetic diversity and phenotypic diversity.** Shown are results for networks that evolved in red noise environments and their corresponding white noise controls averaged over the last 20000 generations of selection (means, +/− S.E.). Upper case letters denote pairwise comparisons among white noise treatments that were significantly different (*P*<0.05, Tukey’s HSD test). Lower case letters denote pairwise comparisons among red noise treatments that were significantly different (*P*<0.05, Tukey’s HSD test). **(A)** Effects on genetic diversity (the mean number of alleles per locus). No effects of period length were detected (main effect, *P* = 0.84; interaction, *P* = 0.29, ANOVA). **(B)** Effects on phenotypic diversity. Period length interacted with noise type (*F*_3,192_ = 7.62, *P*<0.0001, ANOVA).
(TIF)

Figure S8 **Effect of the dominant period length of red noise on evolvability.** Shown are results for networks that evolved in red noise environments and their corresponding white noise controls averaged over the last 20000 generations of selection (means, +/− S.E.). **(A)** Effects on evolvability measured as the rate of adaptation. A significant main effect of evolvability was detected (*F*_3,192_ = 2.8, *P* = 0.044, ANOVA; interaction *P* = 0.68); the rate of adaptation for period = 70 was significantly lower than period = 50 (*P* = 0.025, Tukey’s HSD test; *P*>0.23 all other comparisons). **(B)** Effects on evolvability measured as production of phenotypic variation. No effects of period length were detected (main effect, *P* = 0.19; interaction, *P* = 0.88, ANOVA
(TIF)

Figure S9 **Effects of the dominant period length of red noise on viability epistasis and robustness.** Shown are results for networks that evolved in red noise environments and their corresponding white noise controls averaged over the last 20000 generations of selection (means, +/− S.E.). Upper case letters denote pairwise comparisons among white noise treatments that are significantly different (*P*<0.05, Tukey’s HSD test). Lower case letters denote pairwise comparisons among red noise treatments that are significantly different (*P*<0.05, Tukey’s HSD test). **(A)** Epistatic effects (*β*) of mutations on the percentage of post-mutation phenotypes that were viable. Period length interacted with noise type (*F*_3,192_ = 9.8, *P*<0.0001, ANOVA). **(B)** Viability robustness (*W_1_*) measured as the percentage of networks that were viable under one mutation. Period length interacted with noise type (*F*_3,192_ = 7.6, *P*<0.0001, ANOVA).
(TIF)

Figure S10 **Effects of the dominant period length of red noise on phenotypic epistasis and robustness.** Shown are results for networks that evolved in red noise environments and their corresponding white noise controls averaged over the last 20000 generations of selection (means, +/− S.E.). Upper case letters denote pairwise comparisons among white noise treatments that are significantly different (*P*<0.05, Tukey’s HSD test). Lower case letters denote pairwise comparisons among red noise treatments that are significantly different (*P*<0.05, Tukey’s HSD test). **(A)** Epistatic effects on phenotypic distance (*β*), measured as the Euclidean distance between post- and pre-mutation phenotypes. Period length interacted with noise type (*F*_3,192_ = 14.2, *P*<0.0001, ANOVA). **(B)** Phenotypic robustness (1/*W_1_*) measured as the inverse of phenotypic distance under a single mutation. Period length interacted with noise type (*F*_3,192_ = 25.3, *P*<0.0001, ANOVA).
(TIF)

Figure S11 **Effects of selection regime and reproductive mode (sexual versus asexual) on evolvability, viability robustness and mean log fitness.** Shown are time averages over generations 16000 to 36000 which were then averaged across populations (+/−S.E.). Results for the fluctuating environments are for red noise with a 50 generation period length and its corresponding white noise control. **(A)** Effects on evolvability measured as the rate of adaptation. **(B)** Effects on viability robustness. **(C)** Effects on geometric mean fitness (time-averaged log fitness).
(TIF)

Figure S12 **High resolution time series of dynamics between generations 0 to 500.** Shown are results for networks that evolved in the directional, red noise (period = 50 generations), and white noise (period = 50 generations) environments (means, +/−S.E.). **(A)** Genetic diversity (the mean number of alleles per locus) over time. **(B)** Viability robustness (*W_1_*) over time measured as the percentage of networks that were viable under one mutation. **(C)** Log fitness over time.
(TIF)

Figure S13 **Proportion of matrix elements that have changed over time.** Shown are means across populations (+/−SE) over 36000 generations of selection in the four selection regimes. Results for the fluctuating environments are for red noise with a 50 generation period length and its corresponding white noise control. When analyzing time averages over the last 20000 generations of selection, significant variation among selection regimes was present (*F*_4,120_ = 324.8, *P*<0.0001, ANOVA). Mean matrix change was significantly higher than initial levels for all four selection regimes (*P*<0.001, Tukey’s HSD test).
(TIF)

Figure S14 **Effects of selection regime on the strength and direction of regulatory interactions over time.** Shown are means across populations (+/−SE) over 36000 generations of selection in the four selection regimes. Results for the fluctuating environments are for red noise with a 50 generation period length and its corresponding white noise control. **(A)** Effects on the strength of regulatory interactions. When analyzing time averages over the last 20000 generations of selection, significant variation among selection regimes was present (*F*_4,120_ = 52.5, *P*<0.0001, ANOVA). Interaction strength differed from initial levels only under directional selection (*P*<0.001, Tukey’s HSD test; *P*>0.56, other comparisons). **(B)** Effects on the proportion of positive regulatory interactions. When analyzing time averages over the last 20000 generations of selection, significant variation among selection regimes was present (*F*_4,120_ = 22.6, *P*<0.0001, ANOVA).; values were significantly greater than initial levels only under red noise (*P*<0.0001, Tukey’s HSD test; *P*>0.98, other comparisons).
(TIF)

Figure S15 **The relationship between evolvability and viability robustness and the proportion of positive regulatory interactions.** Shown are population-level means across selection regimes and initial networks. **(A)** Evolvability measured as the rate of adaptation versus the proportion of positive interaction. Shown is the linear regression fit (*R* = 0.42, *P*<0.001, Pearson correlation). **(B)** Viability robustness versus the proportion of positive interaction. Shown is the linear regression fit (*R* = 0.28, *P* = 0.016, Pearson correlation). Symbols as in Fig. S3.
(TIF)
