# Supplementary figures and images for: Environmental Noise, Genetic Diversity and the Evolution of Evolvability and Robustness in Model Gene Networks
Source: PLoS One. 2012 Dec 20;7(12):e52204. doi: 10.1371/journal.pone.0052204 (PMC3527431; doi:10.1371/journal.pone.0052204)

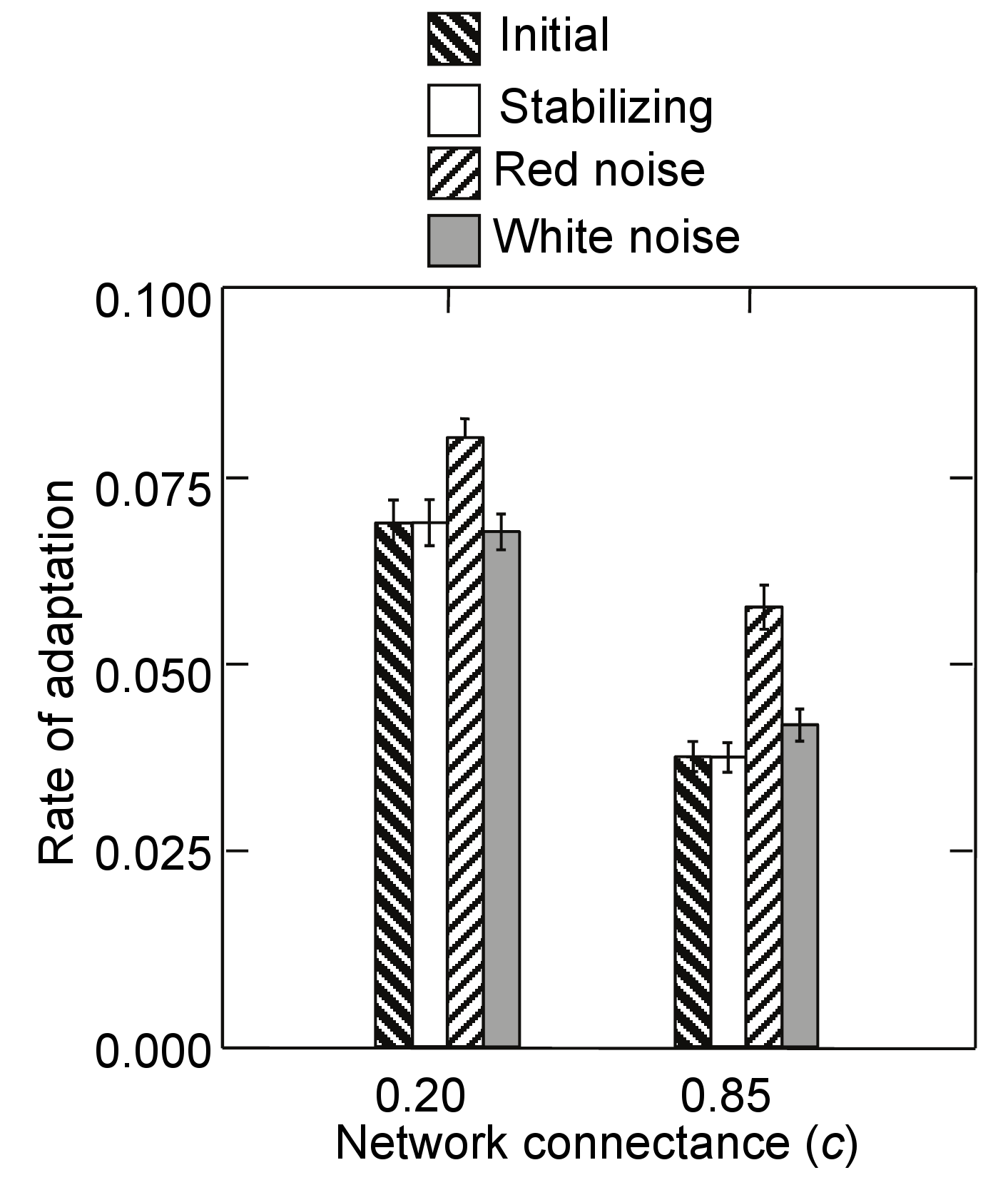

Supplement: Figure S1 — Effects of selection on evolvability (the rate of adaptation) for networks with 0.20 and 0.80 connectance (c). Results were measured after 2000 generations of selection on 25 randomly generated initial networks. Results for the fluctuating environments are for red noise with a 50 generation period length and its corresponding white noise control. Shown are means +/−S.E. (TIF) [file pone.0052204.s001.tif]

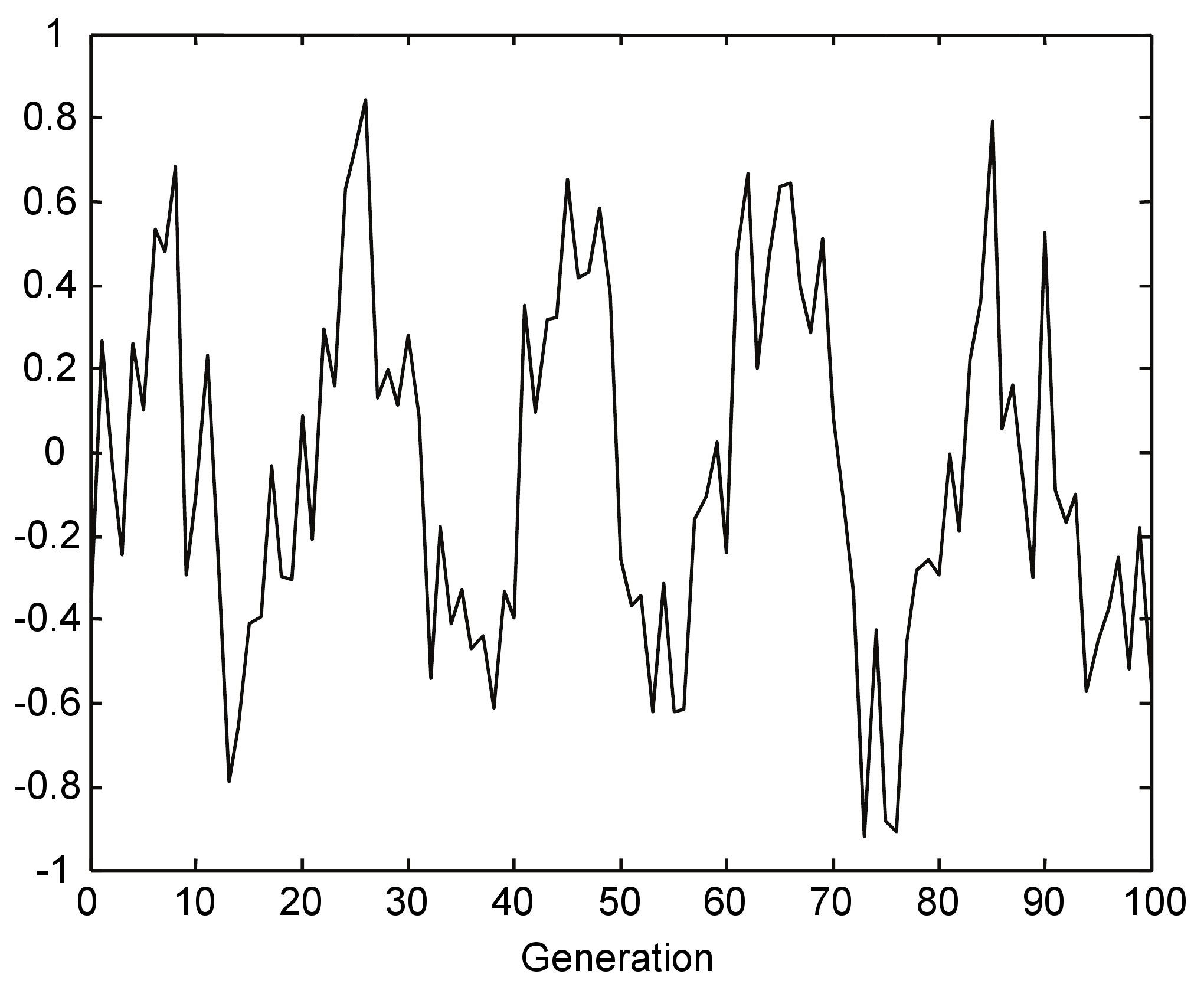

Supplement: Figure S2 — An example of a red noise time series. Results were generated using equation 4, with a period length of the largest amplitude fluctuation equal to 20 generations, a time step of one generation, and g = 1. (TIF) [file pone.0052204.s002.tif]

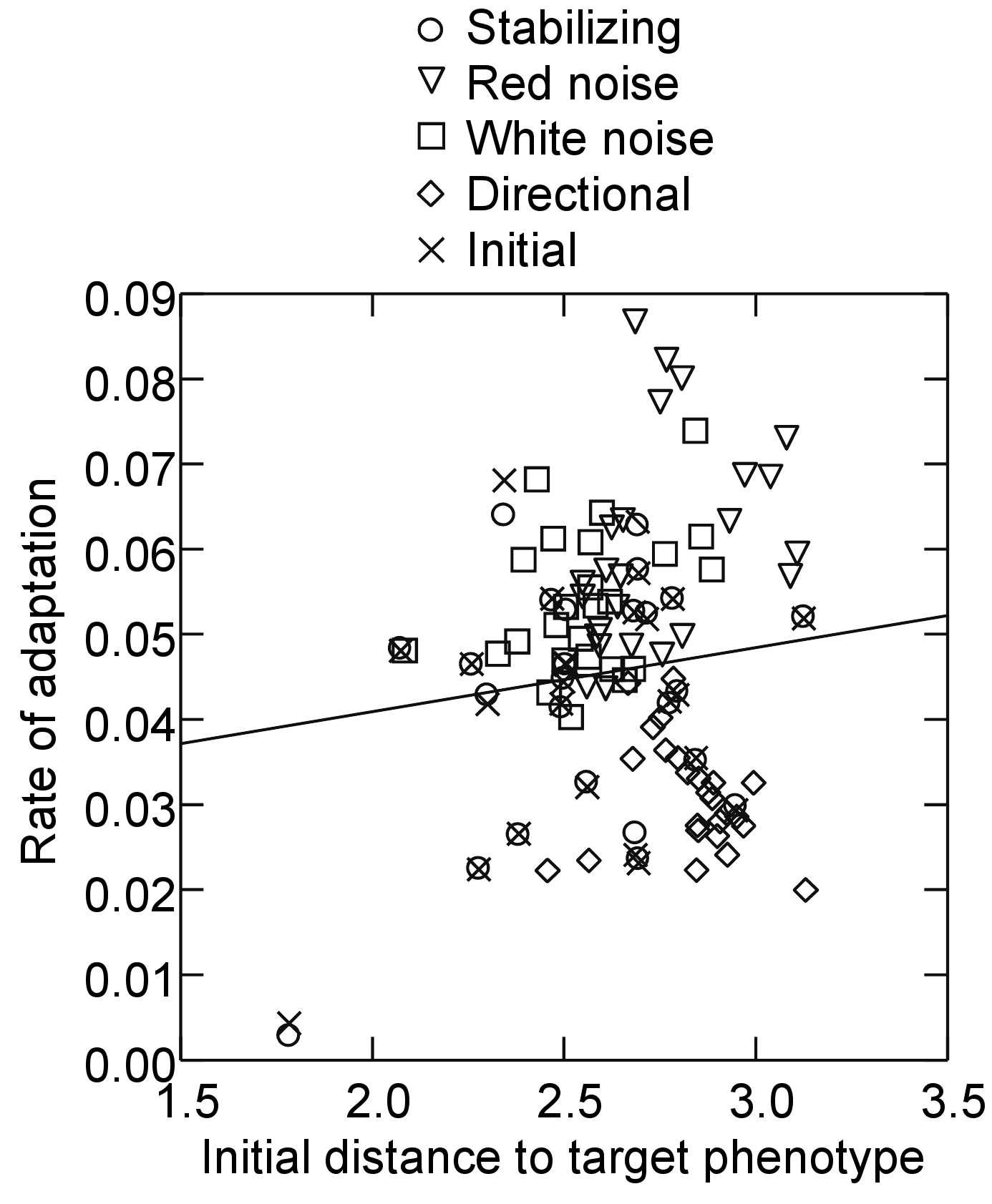

Supplement: Figure S3 — The relationship between the initial distance to evolvability target phenotypes and evolvability measured as the rate of adaptation. Results are population means (+/− S.E.). Shown is the linear regression fit. (TIF) [file pone.0052204.s003.tif]

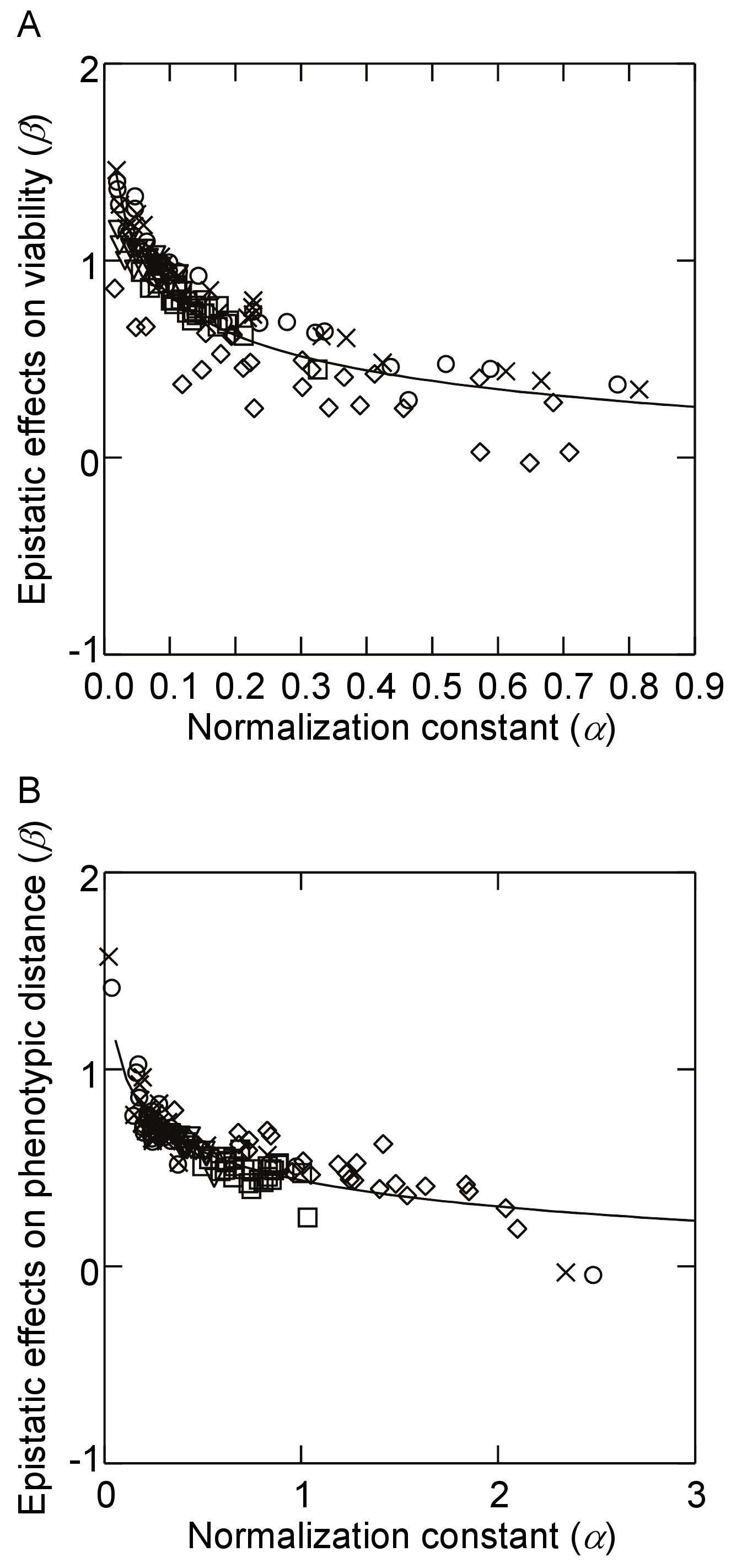

Supplement: Figure S4 — The relationship between epistatic effects ( β ) and the normalization constant ( α ). (A) Results based on mutation effects on log percent viability. (B) Results based on mutation effects on phenotypic distance. Shown is the fit for the power relationship b = −aαz+σ. Data are from the initial networks and time-averages over the last 20000 generations of selection for four selection regimes. Results for the fluctuating environments are for red noise with a 50 generation period length and its corresponding white noise control. Symbols as in Fig. S3. (TIF) [file pone.0052204.s004.tif]

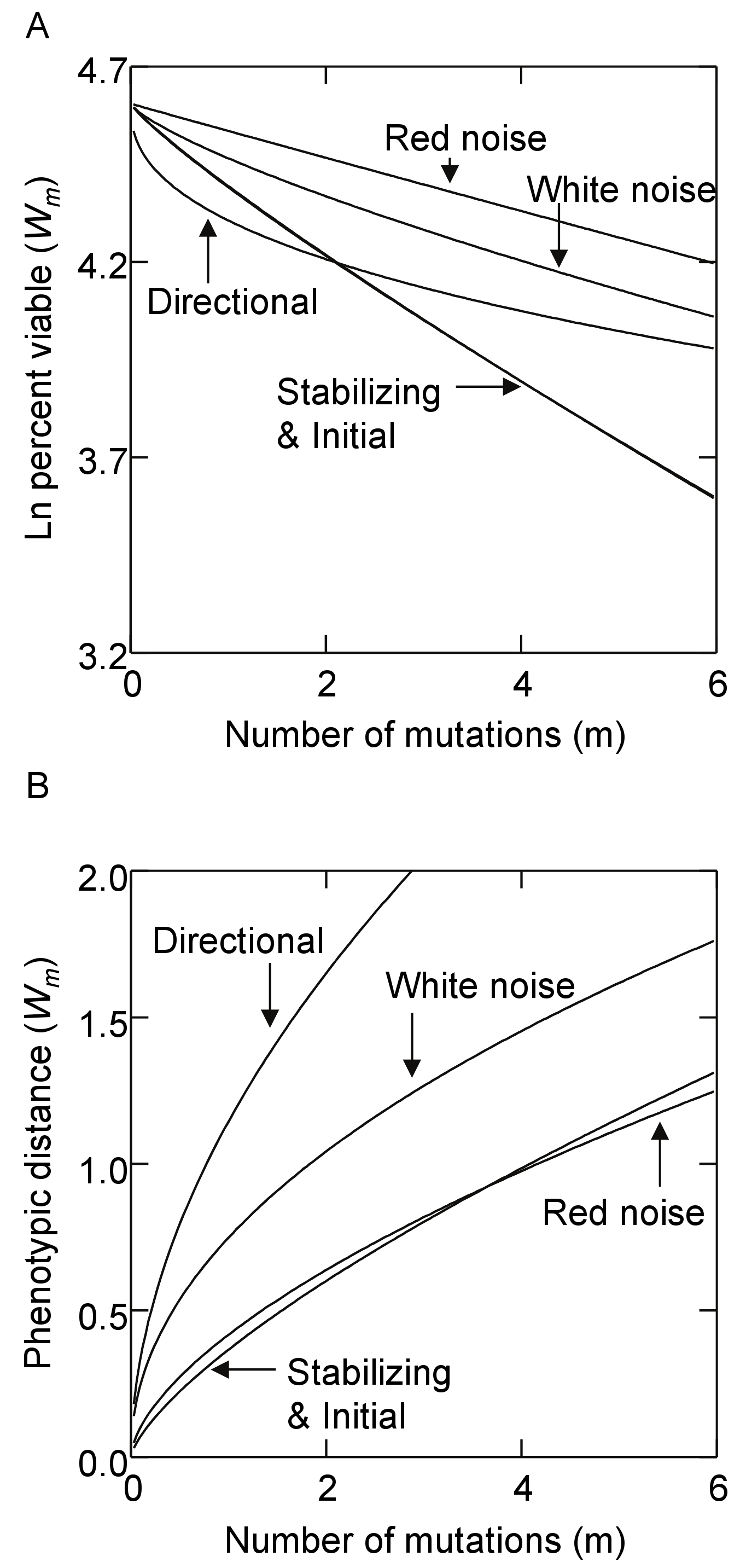

Supplement: Figure S5 — Mutation effects on components of fitness. Separate relationships are shown for the initial (pre-selection) networks and networks that experienced selection under stabilizing, directional, red noise (period = 50 generations) and white noise (period = 50 generations) selection. (A) The relationship between the number of mutations (m) and the natural log of the percentage of networks that are viable (Wm). Curves were generated using the relationship ln(Wm) = −αmβ+ ln(100). (B) The relationship between the number of mutations (m) and the phenotypic distance between mutant and pre-mutation phenotypes (Wm). Curves were generated using the relationship Wm = αmβ. In the case of networks under selection, α and β were first estimated as time-averages from model results over the last 20000 generations then averaged across populations. (TIF) [file pone.0052204.s005.tif]

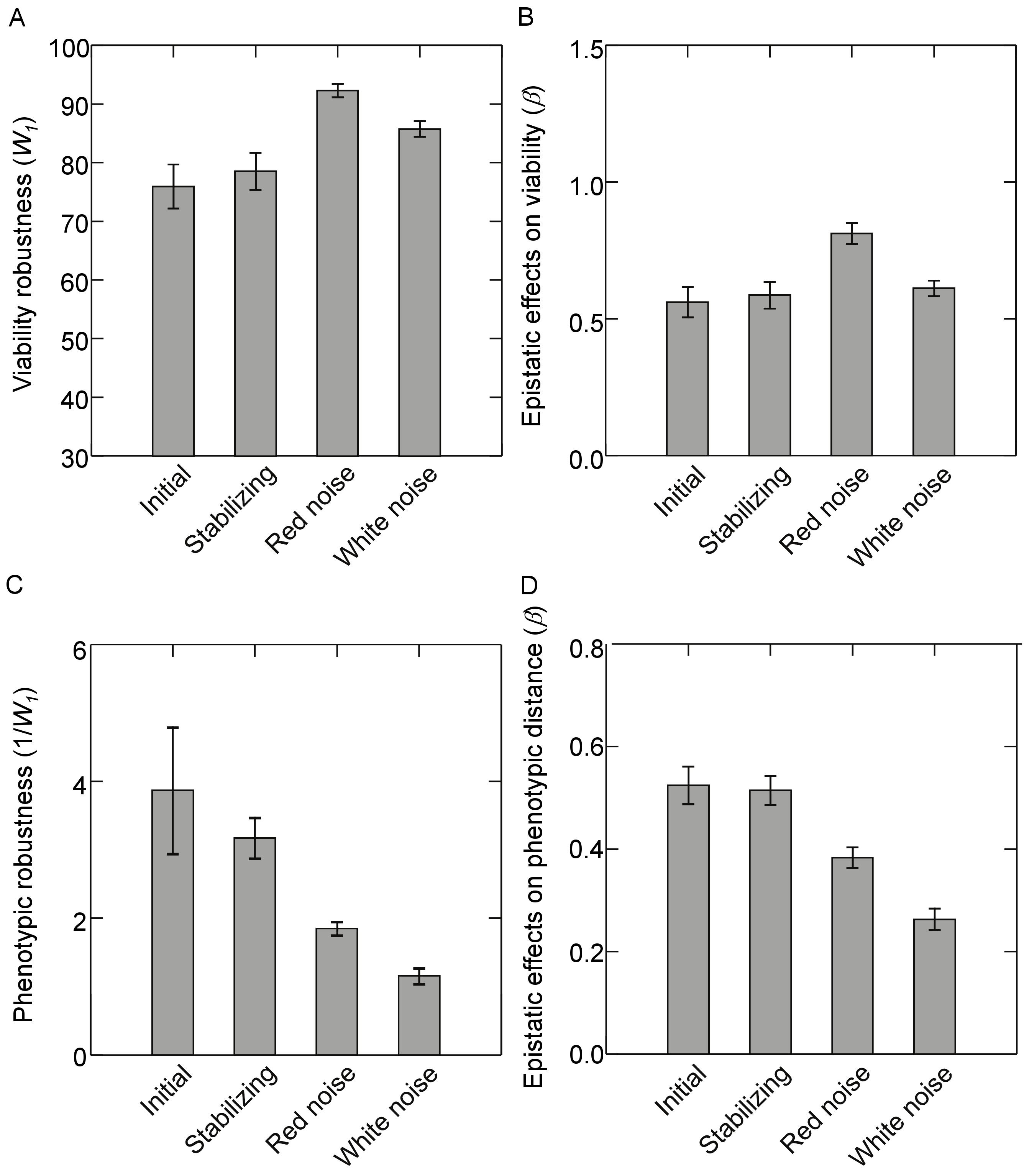

Supplement: Figure S6 — Effects of selection on epistasis and robustness measured using an expanded range of mutations (1–50). Shown are results for the initial (pre-selection) networks and networks after 36000 generations under stabilizing, directional, red noise (period = 50 generations) and white noise (period = 50 generations) selection (means, +/−S.E.). (A) Effects on viability robustness (W1). A significant effect of environment was detected (F 3,96 = 8.4, P<0.001, ANOVA); robustness of red and white noise networks was significantly greater than initial levels (P<0.001, Tukey’s HSD test). (B) Effects on epistatic effects on viability (β). A significant effect of environment was detected (F 3,96 = 7.1, P<0.001, ANOVA); β of red noise networks was significantly greater than initial levels (P<0.001, Tukey’s HSD test; P = 0.84 for white noise versus initial). (C) Effects on phenotypic robustness (1/W1). A significant effect of environment was detected (F 3,96 = 6.6, P<0.001, ANOVA); robustness of red and white noise networks was significantly lower than initial levels (P<0.02, Tukey’s HSD test). (D) Effects on epistatic effects on phenotypic distance (β). A significant effect of environment was detected (F 3,96 = 21.3, P<0.001, ANOVA); β in red and white noise networks was significantly lower than initial levels (P<0.01, Tukey’s HSD test). (TIF) [file pone.0052204.s006.tif]

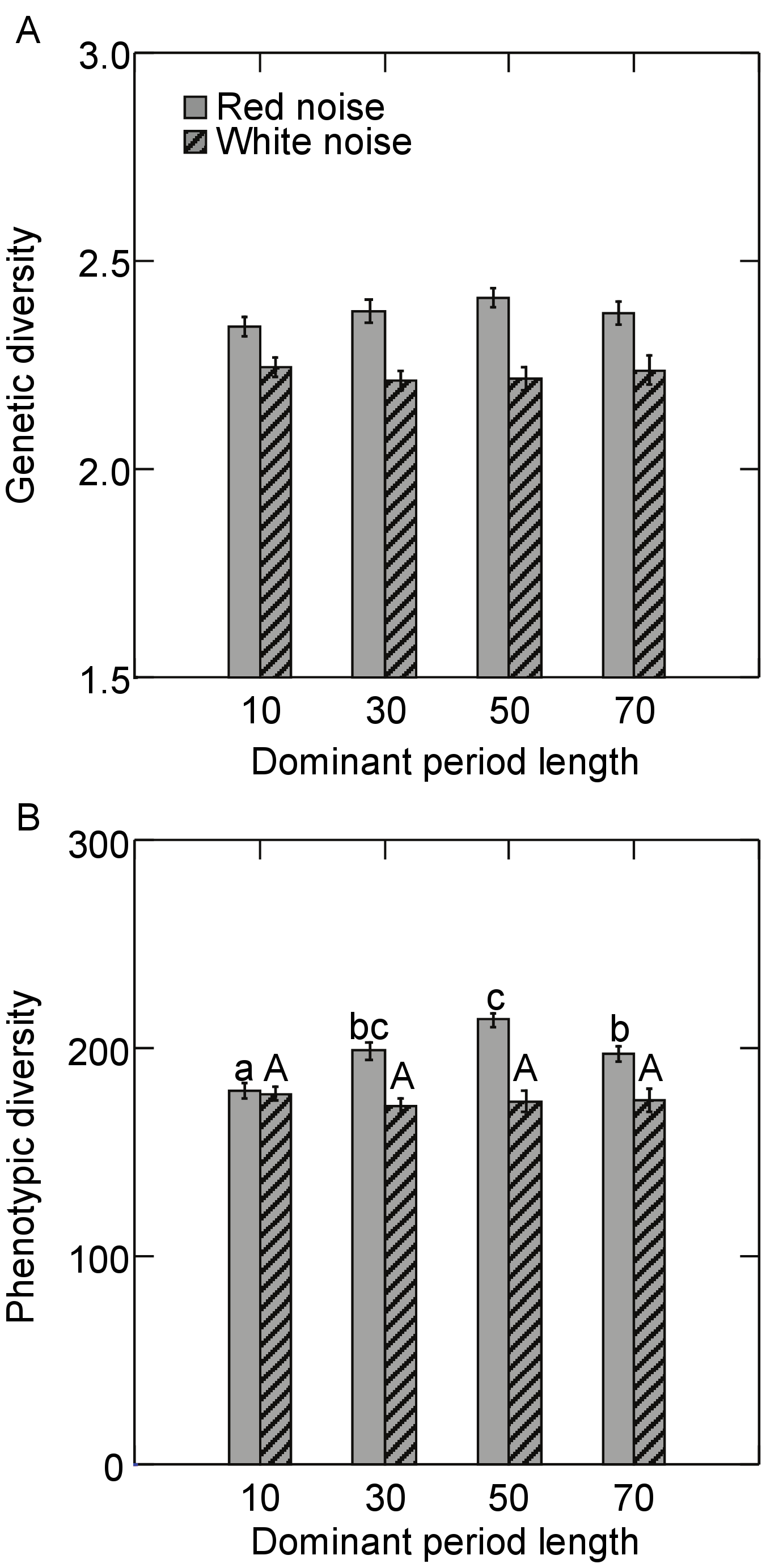

Supplement: Figure S7 — Effect of the dominant period length of red noise on genetic diversity and phenotypic diversity. Shown are results for networks that evolved in red noise environments and their corresponding white noise controls averaged over the last 20000 generations of selection (means, +/− S.E.). Upper case letters denote pairwise comparisons among white noise treatments that were significantly different (P<0.05, Tukey’s HSD test). Lower case letters denote pairwise comparisons among red noise treatments that were significantly different (P<0.05, Tukey’s HSD test). (A) Effects on genetic diversity (the mean number of alleles per locus). No effects of period length were detected (main effect, P = 0.84; interaction, P = 0.29, ANOVA). (B) Effects on phenotypic diversity. Period length interacted with noise type (F 3,192 = 7.62, P<0.0001, ANOVA). (TIF) [file pone.0052204.s007.tif]

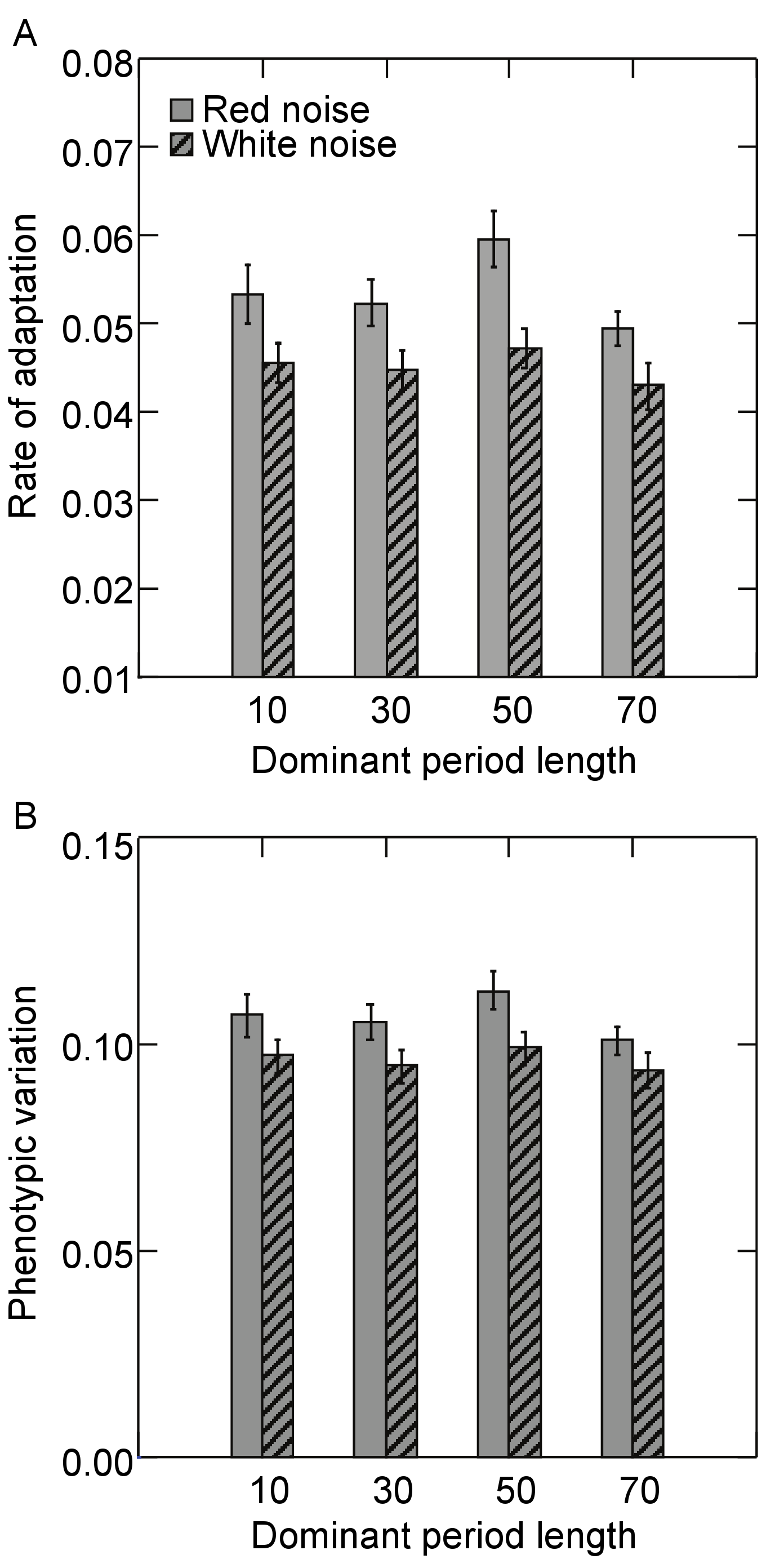

Supplement: Figure S8 — Effect of the dominant period length of red noise on evolvability. Shown are results for networks that evolved in red noise environments and their corresponding white noise controls averaged over the last 20000 generations of selection (means, +/− S.E.). (A) Effects on evolvability measured as the rate of adaptation. A significant main effect of evolvability was detected (F 3,192 = 2.8, P = 0.044, ANOVA; interaction P = 0.68); the rate of adaptation for period = 70 was significantly lower than period = 50 (P = 0.025, Tukey’s HSD test; P>0.23 all other comparisons). (B) Effects on evolvability measured as production of phenotypic variation. No effects of period length were detected (main effect, P = 0.19; interaction, P = 0.88, ANOVA (TIF) [file pone.0052204.s008.tif]

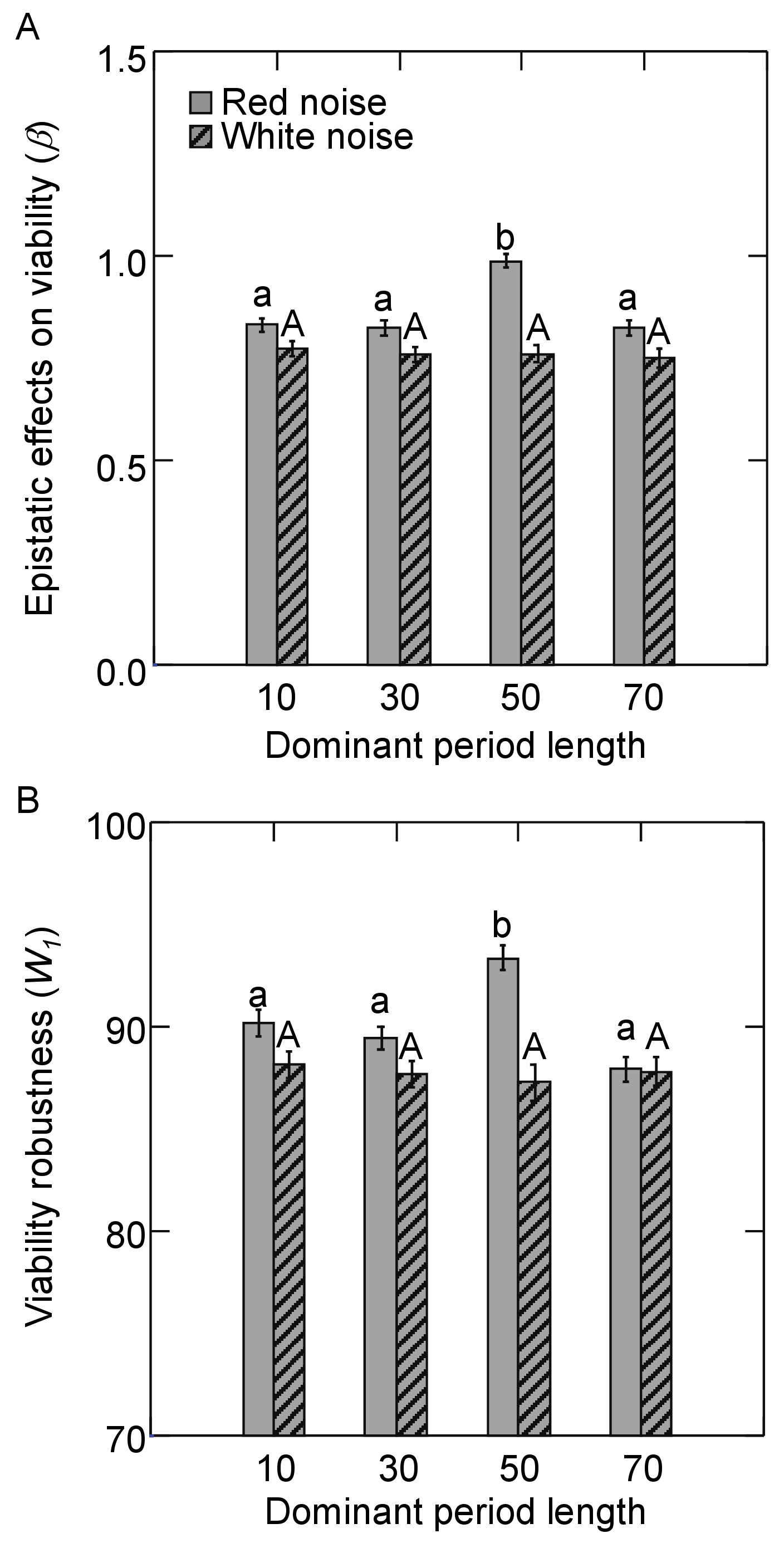

Supplement: Figure S9 — Effects of the dominant period length of red noise on viability epistasis and robustness. Shown are results for networks that evolved in red noise environments and their corresponding white noise controls averaged over the last 20000 generations of selection (means, +/− S.E.). Upper case letters denote pairwise comparisons among white noise treatments that are significantly different (P<0.05, Tukey’s HSD test). Lower case letters denote pairwise comparisons among red noise treatments that are significantly different (P<0.05, Tukey’s HSD test). (A) Epistatic effects (β) of mutations on the percentage of post-mutation phenotypes that were viable. Period length interacted with noise type (F 3,192 = 9.8, P<0.0001, ANOVA). (B) Viability robustness (W1) measured as the percentage of networks that were viable under one mutation. Period length interacted with noise type (F 3,192 = 7.6, P<0.0001, ANOVA). (TIF) [file pone.0052204.s009.tif]

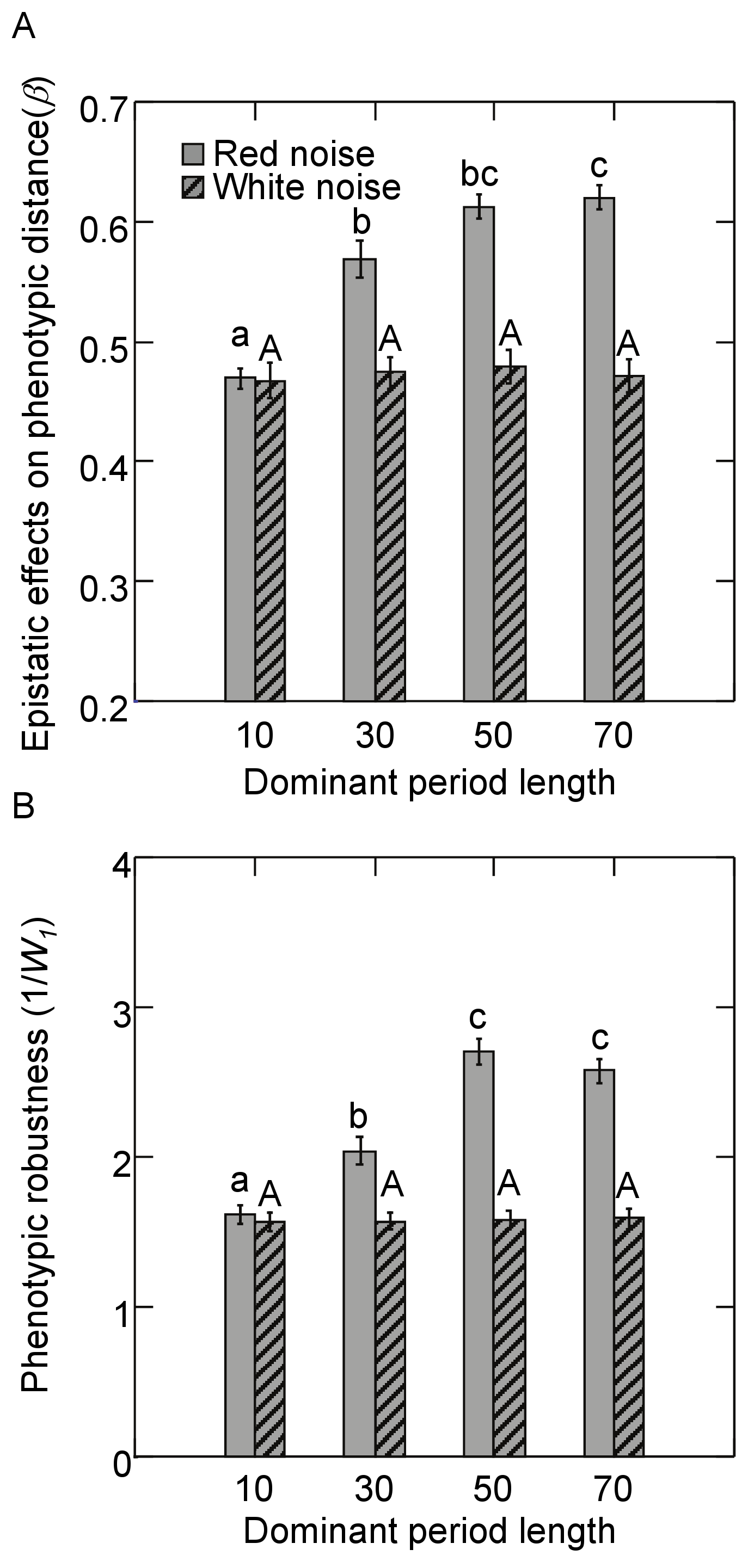

Supplement: Figure S10 — Effects of the dominant period length of red noise on phenotypic epistasis and robustness. Shown are results for networks that evolved in red noise environments and their corresponding white noise controls averaged over the last 20000 generations of selection (means, +/− S.E.). Upper case letters denote pairwise comparisons among white noise treatments that are significantly different (P<0.05, Tukey’s HSD test). Lower case letters denote pairwise comparisons among red noise treatments that are significantly different (P<0.05, Tukey’s HSD test). (A) Epistatic effects on phenotypic distance (β), measured as the Euclidean distance between post- and pre-mutation phenotypes. Period length interacted with noise type (F 3,192 = 14.2, P<0.0001, ANOVA). (B) Phenotypic robustness (1/W1) measured as the inverse of phenotypic distance under a single mutation. Period length interacted with noise type (F 3,192 = 25.3, P<0.0001, ANOVA). (TIF) [file pone.0052204.s010.tif]

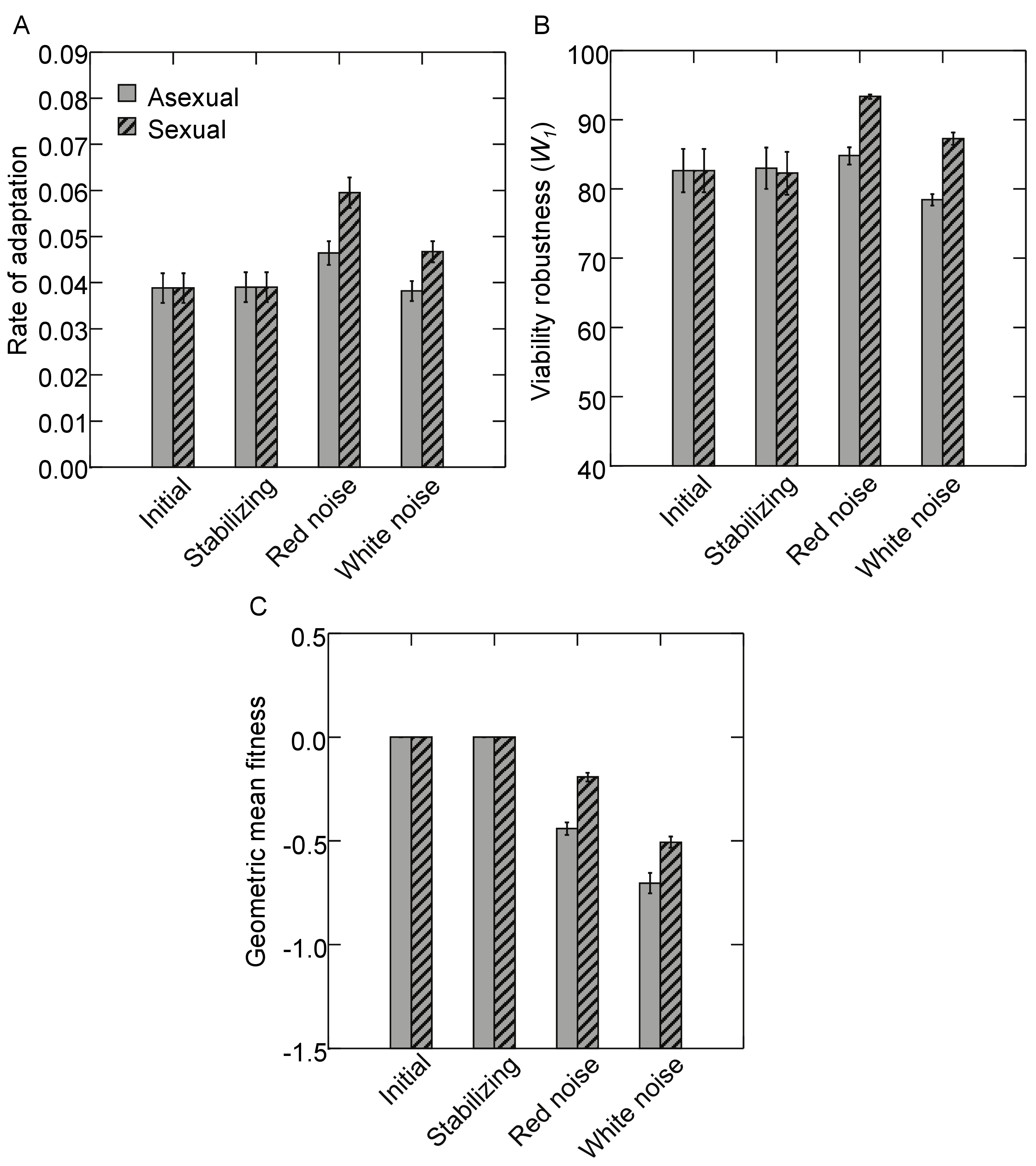

Supplement: Figure S11 — Effects of selection regime and reproductive mode (sexual versus asexual) on evolvability, viability robustness and mean log fitness. Shown are time averages over generations 16000 to 36000 which were then averaged across populations (+/−S.E.). Results for the fluctuating environments are for red noise with a 50 generation period length and its corresponding white noise control. (A) Effects on evolvability measured as the rate of adaptation. (B) Effects on viability robustness. (C) Effects on geometric mean fitness (time-averaged log fitness). (TIF) [file pone.0052204.s011.tif]

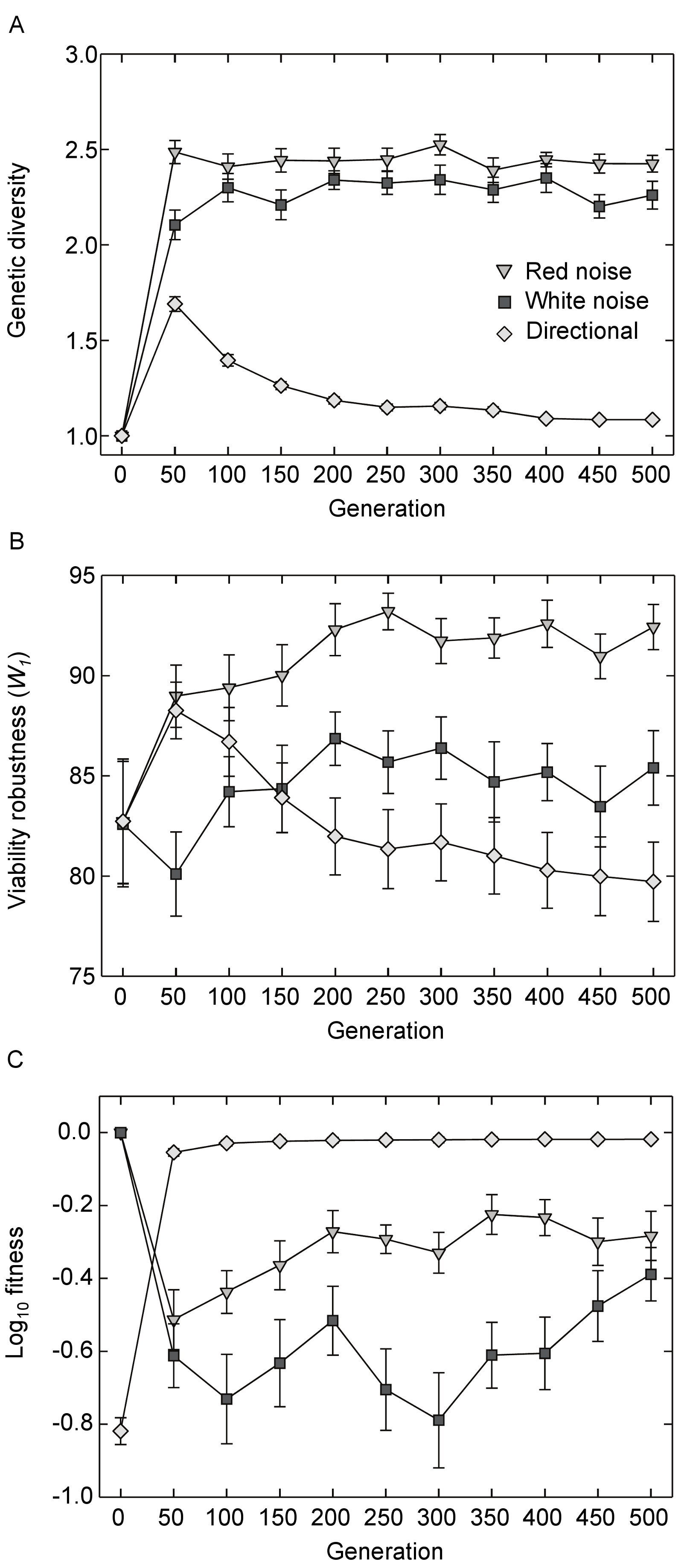

Supplement: Figure S12 — High resolution time series of dynamics between generations 0 to 500. Shown are results for networks that evolved in the directional, red noise (period = 50 generations), and white noise (period = 50 generations) environments (means, +/−S.E.). (A) Genetic diversity (the mean number of alleles per locus) over time. (B) Viability robustness (W1) over time measured as the percentage of networks that were viable under one mutation. (C) Log fitness over time. (TIF) [file pone.0052204.s012.tif]

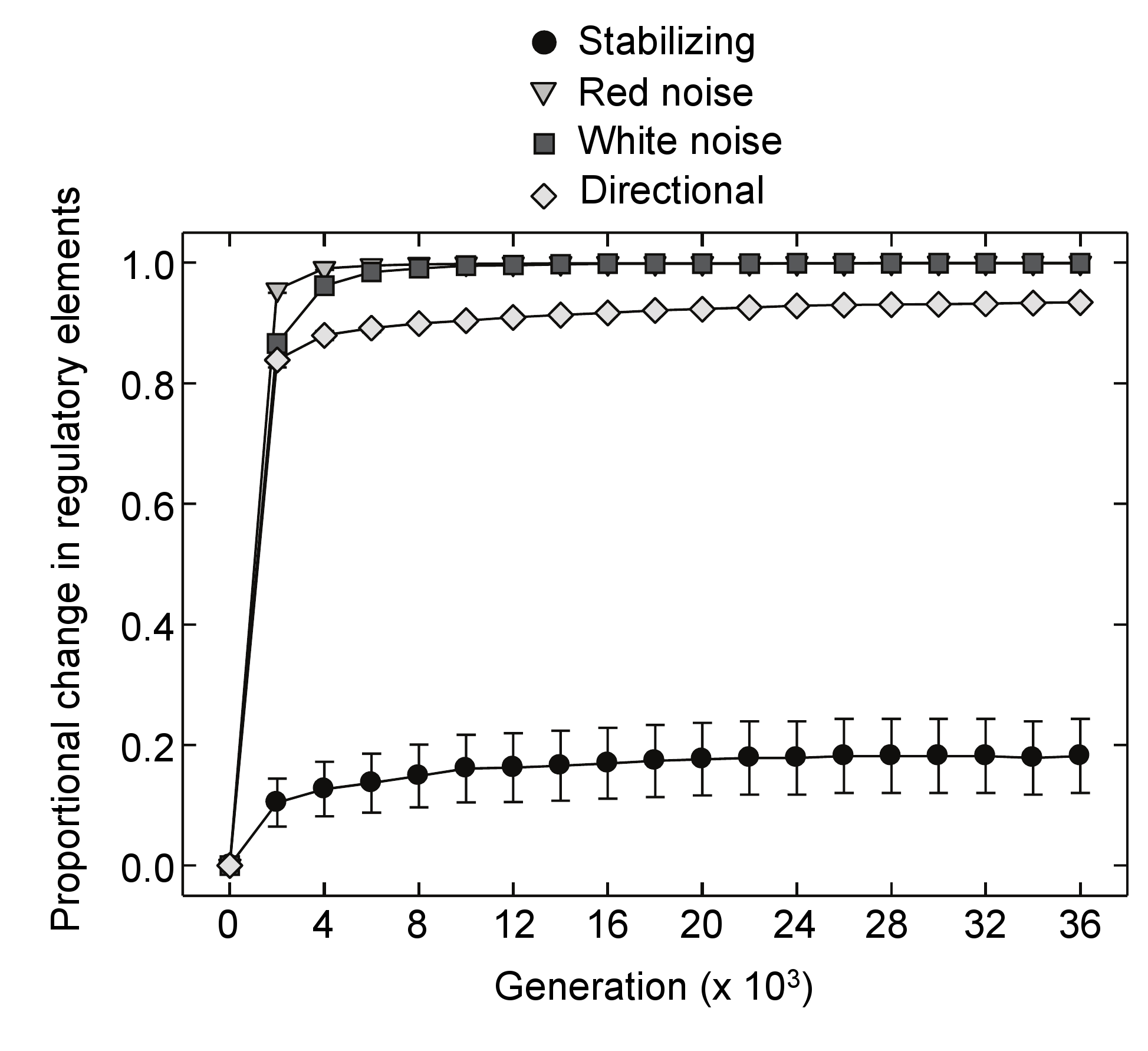

Supplement: Figure S13 — Proportion of matrix elements that have changed over time. Shown are means across populations (+/−SE) over 36000 generations of selection in the four selection regimes. Results for the fluctuating environments are for red noise with a 50 generation period length and its corresponding white noise control. When analyzing time averages over the last 20000 generations of selection, significant variation among selection regimes was present (F 4,120 = 324.8, P<0.0001, ANOVA). Mean matrix change was significantly higher than initial levels for all four selection regimes (P<0.001, Tukey’s HSD test). (TIF) [file pone.0052204.s013.tif]

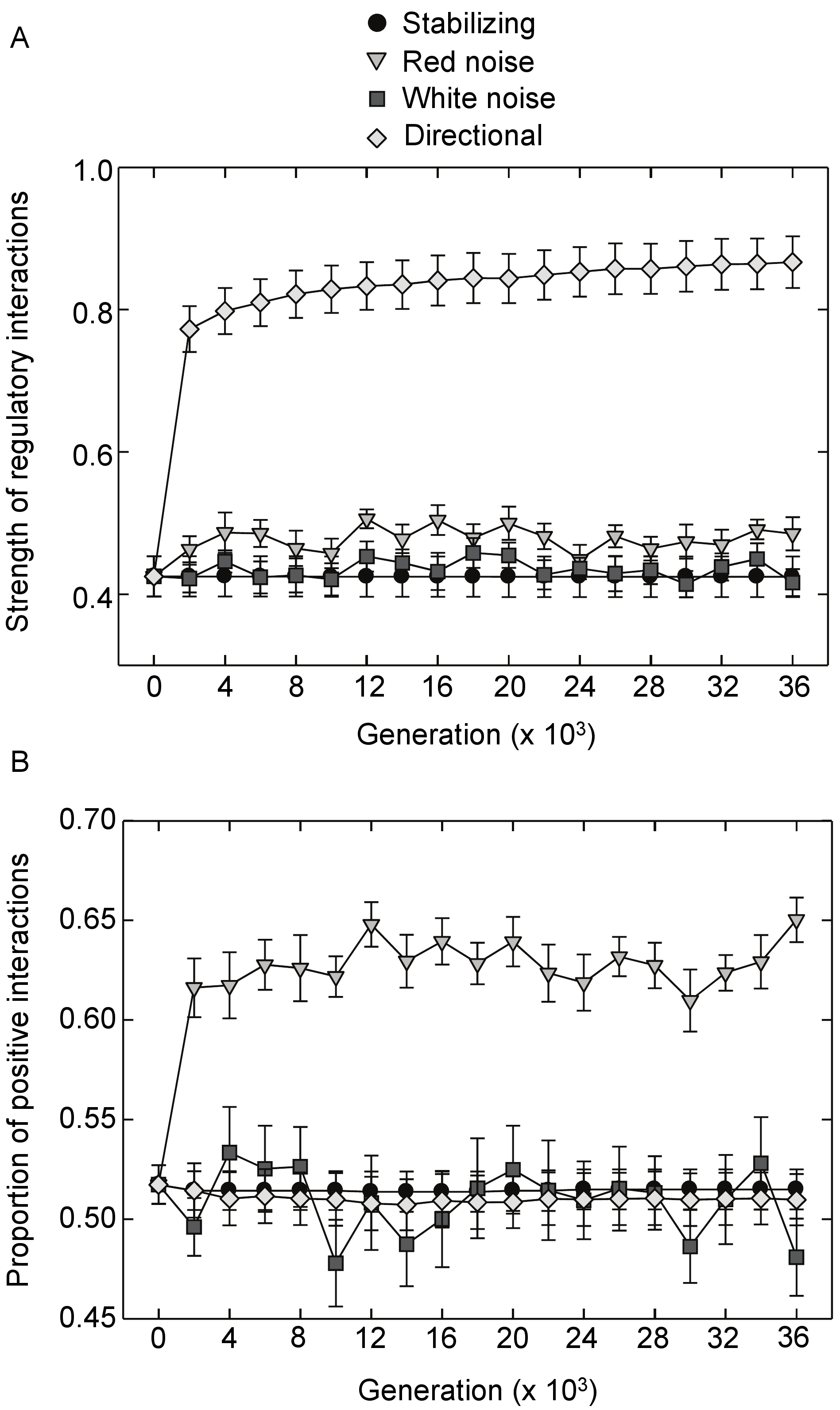

Supplement: Figure S14 — Effects of selection regime on the strength and direction of regulatory interactions over time. Shown are means across populations (+/−SE) over 36000 generations of selection in the four selection regimes. Results for the fluctuating environments are for red noise with a 50 generation period length and its corresponding white noise control. (A) Effects on the strength of regulatory interactions. When analyzing time averages over the last 20000 generations of selection, significant variation among selection regimes was present (F 4,120 = 52.5, P<0.0001, ANOVA). Interaction strength differed from initial levels only under directional selection (P<0.001, Tukey’s HSD test; P>0.56, other comparisons). (B) Effects on the proportion of positive regulatory interactions. When analyzing time averages over the last 20000 generations of selection, significant variation among selection regimes was present (F 4,120 = 22.6, P<0.0001, ANOVA).; values were significantly greater than initial levels only under red noise (P<0.0001, Tukey’s HSD test; P>0.98, other comparisons). (TIF) [file pone.0052204.s014.tif]

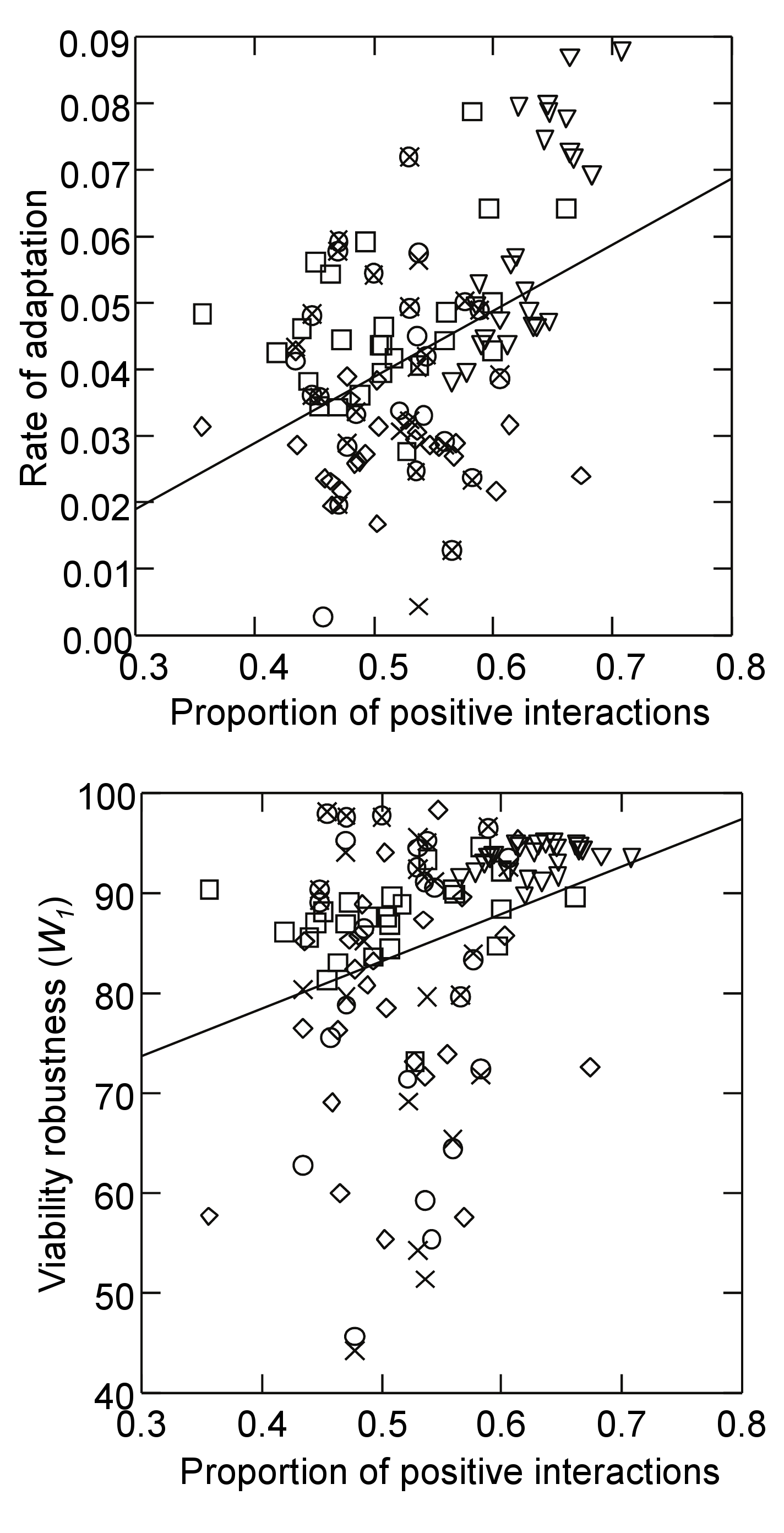

Supplement: Figure S15 — The relationship between evolvability and viability robustness and the proportion of positive regulatory interactions. Shown are population-level means across selection regimes and initial networks. (A) Evolvability measured as the rate of adaptation versus the proportion of positive interaction. Shown is the linear regression fit (R = 0.42, P<0.001, Pearson correlation). (B) Viability robustness versus the proportion of positive interaction. Shown is the linear regression fit (R = 0.28, P = 0.016, Pearson correlation). Symbols as in Fig. S3. (TIF) [file pone.0052204.s015.tif]
